# Adaptive Inverse Control Using the Krasnosel’skii-Pokrovskii Model for Hysteresis Compensation in Piezoelectric Flexure Micro-Positioning Stage

**DOI:** 10.3390/mi17080917

**Published:** 2026-07-30

**Authors:** Yuansheng Chen, Hao Lou, Jian Wang, Shaona Liu

**Affiliations:** 1 School of Mechanical Engineering, Yancheng Institute of Technology, Yancheng 224051, China; chenys@ycit.edu.cn (Y.C.); 19827066706@163.com (H.L.); 2 School of Energy and Power Engineering, Nanjing University of Science and Technology, Nanjing 210094, China; wjnjust801@163.com

**Keywords:** piezoelectric flexure micro-positioning stage, straight circular flexure hinge, two-stage displacement amplifier, Krasnosel’skii-Pokrovskii hysteresis model, adaptive inverse control, hysteresis compensation

## Abstract

Piezoelectric flexure micro-positioning stages are essential micromotion actuators for micro-assembly, atomic force microscopy and nano-manufacturing, but intrinsic hysteresis nonlinearity of piezoelectric stacks distorts the linear voltage-to-displacement mapping and induces significant micro-positioning errors. Conventional hysteresis compensation based on offline-calibrated Krasnosel’skii-Pokrovskii (KP) models cannot adapt to time-varying excitation, whereas state-of-the-art adaptive KP control requires auxiliary dynamic equations and imposes high computational overhead on miniature real-time controllers. To address these limitations, this paper develops a single-degree-of-freedom micromotion positioning device equipped with symmetric two-stage displacement amplification mechanisms and straight circular flexure hinges. ANSYS finite element simulations validate the mechanical stiffness, structural safety and linear amplification characteristic of the micro-positioning stage, achieving a maximum output stroke of 95.95 μm. A discretized KP hysteresis model is constructed to accurately capture the asymmetric rate-dependent hysteresis of piezoelectric stacks. On this basis, a lightweight adaptive inverse control framework is proposed, which realizes online tuning of KP weights through gradient descent iteration only relying on real-time position feedback, eliminating static pre-calibration and extra dynamic correction links. Tracking experiments under 0.1–2 Hz sinusoidal waveforms and 3–7 V variable-amplitude sinusoidal waveforms are implemented. Experimental results show that the proposed approach reduces the root-mean-square error (RMSE) by 7.41–85.65% and the mean absolute percentage error (MAPE) by 7.56–87.81% compared with uncompensated open-loop micromotion control. The combined micro-flexure mechanical design and adaptive hysteresis compensation strategy greatly improves positioning accuracy and anti-interference capacity, offering a low-computation technical route for high-performance micro-positioning systems.

## 1. Introduction

Driven by micro-nano manufacturing and ultra-precision positioning demands, piezoelectric flexure micro-positioning stages have become core actuators for atomic force microscopy, micro-assembly and nano-processing, benefiting from compact structure, nanometer resolution and fast response. Because it still requires measured input and output data before control experiment, the incorporation of KP model and adaptive control was only validated in open-loop control, which is also a guidance for future research on hysteresis compensation [1,2,3]. Advanced composite piezoelectric materials with excellent deformation capacity provide new ideas for micro-drive design [4]. However, inherent voltage-displacement hysteresis nonlinearity of piezoelectric stacks introduces large positioning deviation and weakens dynamic stability, which restricts high-precision operation and has become a hot research topic in precision actuation.

### 1.1. Research Progress of Piezoelectric Micro-Positioning Stages

**Figure 1:**
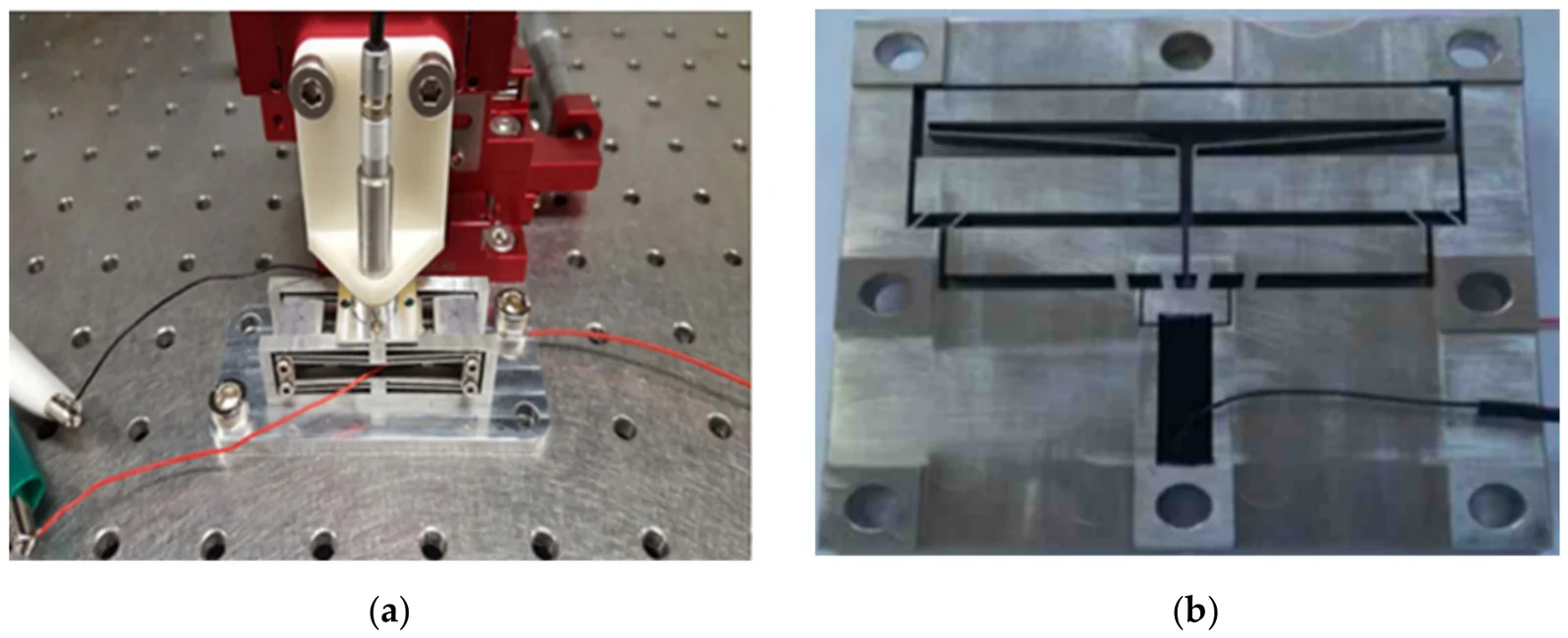
One-dimensional micro-positioning stage. (**a**) Vertical motion micro-positioning stage; (**b**) Micro-positioning stage based on cross hinges [5,6].

In the single-degree-of-freedom piezoelectric flexure micro-positioning stage, Wu et al. designed a compact stage in the vertical direction, using orthogonal composite bridge amplifiers, with a displacement resolution of 10 nm and a maximum travel of 97.32 μm [5]. As shown in Figure 1a. Jung et al. proposed a multi-bar motion stage based on a new type of cross hinge, with an amplification ratio of up to 60 times, achieving 20 nm step positioning, breaking through the performance limitations of traditional hinges [6]. As shown in Figure 1b. A variety of single-degree-of-freedom flexure amplification structures with balanced stroke and linearity have been reported recently [7].

**Figure 2:**
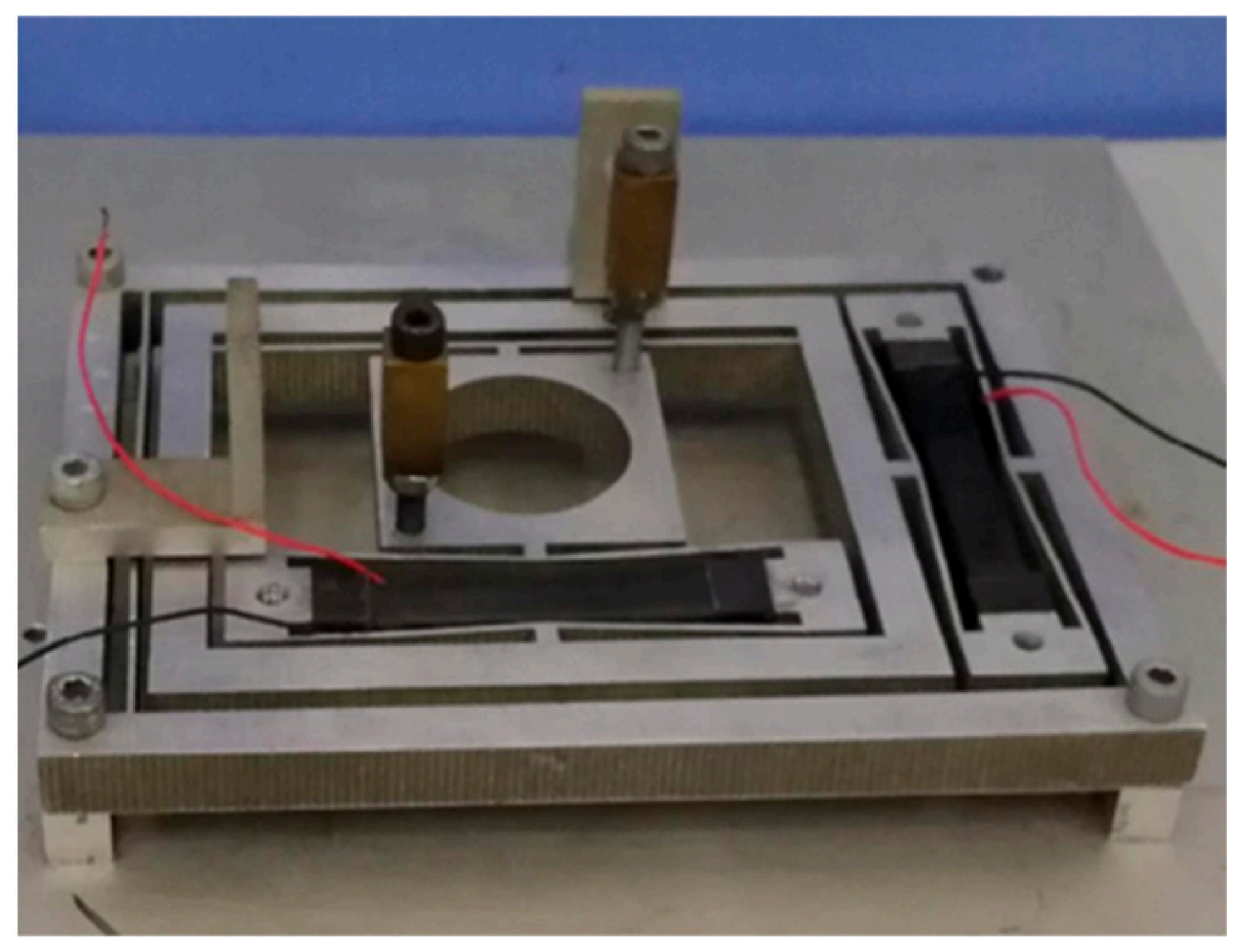
Equal stiffness and equal stroke X-Y two-dimensional micro-positioning stage [8].

In the two-dimensional micro-positioning stage, Sun et al. proposed an X-Y stage with equal stiffness and equal stroke [8]. Through a sand hourglass-type amplification mechanism and a nested structure, the displacement coupling was reduced. The maximum stroke of the X and Y axes was approximately 500 μm. The prototype of this X-Y positioning stage is shown in Figure 2.

**Figure 3:**
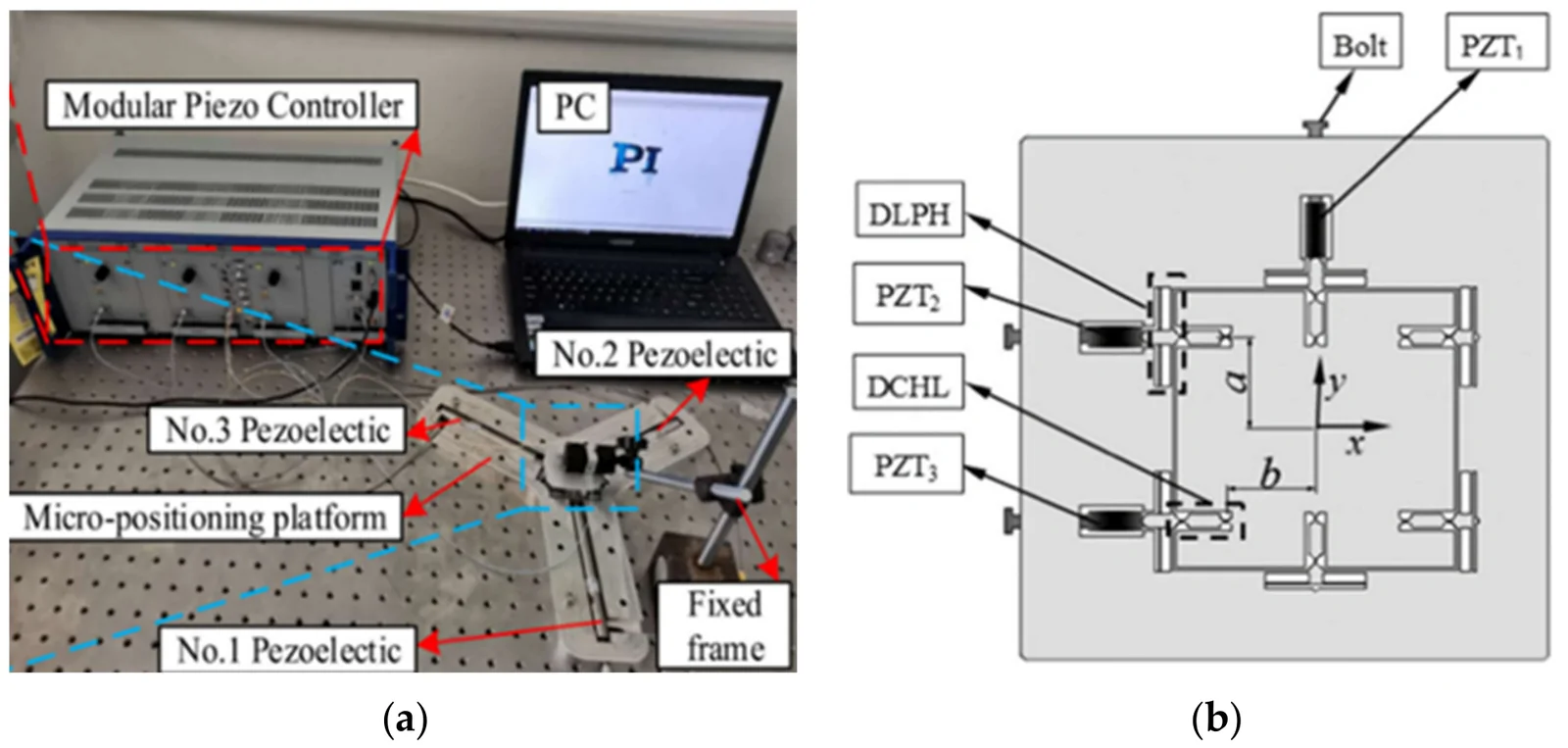
Three-dimensional micro-positioning stage. (**a**) High-precision rigid-flexible coupling three-dimensional micro-positioning stage; (**b**) An X-Y-θ micro-positioning stage based on flexible hinges [9,10].

The three-dimensional micro-positioning stage is mainly divided into two types, X-Y-Z linear motion and X-Y-θ combined motion. Wang et al. proposed a rigid-flexible coupling stage, which uses symmetrical straight circular flexure hinges and wedge-shaped block mechanisms to achieve a displacement output of 32.4 μm × 25.5 μm × 40.2 μm [9]. The tracking accuracy of the X/Y/Z axes is 12 nm, 7 nm, and 60 nm respectively. As shown in Figure 3a. Guo et al. designed a three-degree-of-freedom stage, which uses orthogonal configuration piezoelectric actuators to achieve decoupled motion [10]. The maximum travel of the X/Y axes exceeds 12 μm. As shown in Figure 3b.

**Figure 4:**
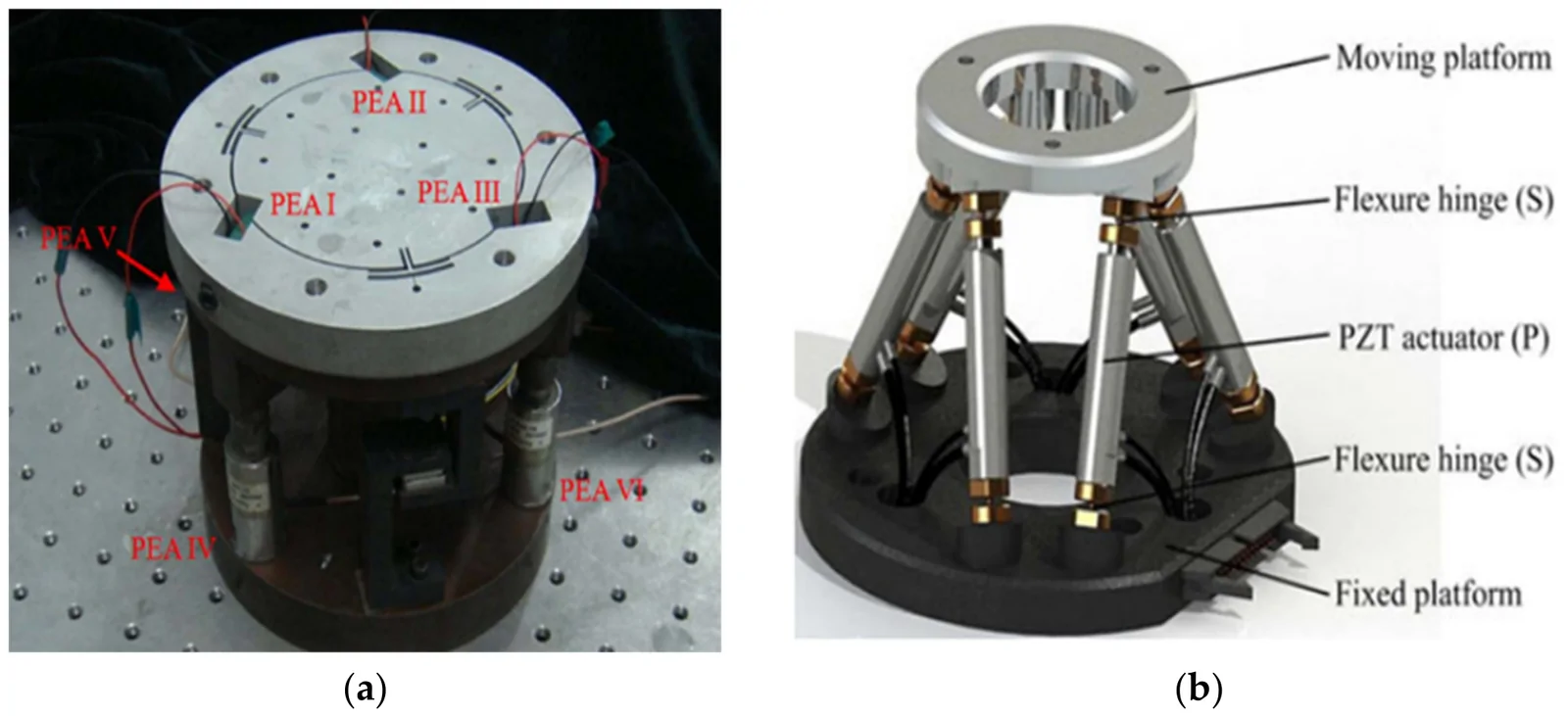
Six-degree-of-freedom micro-positioning stage. (**a**) New six-degree-of-freedom positioning system; (**b**) Piezoelectric high-precision flexible parallel pointing stage [11,12].

In terms of the six-degree-of-freedom positioning system, Cai et al. combined two three-degree-of-freedom stages to achieve a spatial travel of 8.2 mm × 10.5 mm × 13.0 mm and a rotation range of within 224 μrad [11]. The static and dynamic characteristics were stable. As shown in Figure 4a. Du et al. developed a satellite optical communication stage, which used a straight circular flexure hinge as the rotational joint [12]. The linear travel was ±58 μm × ±50 μm × 40 μm, the rotation range was ±421.9 μrad × ±449.2 μrad × ±2007.9 μrad, the steering resolution was less than 0.1μrad, and there was no backlash or friction interference. As shown in Figure 4b.

The existing micro-positioning stages have made significant progress in terms of travel, accuracy and degrees of freedom, but still have shortcomings. Single-degree-of-freedom piezoelectric flexure micro-positioning stages are unable to meet complex motion requirements, while multi-degree-of-freedom stages suffer from issues such as displacement coupling and complex structures. Moreover, most of these stages do not fully consider the impact of hysteresis nonlinearity on positioning accuracy, and there is an urgent need to combine efficient drive control strategies to enhance performance.

### 1.2. Hysteresis Modeling and Model-Based Control Strategies

Hysteresis modeling methods can be classified into three categories: physical models, phenomenological models, and intelligent models. Physical models start with the microscopic mechanism of materials. The Jiles-Atherton model is based on the domain wall theory of ferromagnetic materials and requires many parameters and is only applicable to specific materials [13,14]. The Maxwell model describes mechanical lag via a spring-damper system but can only fit symmetric lag curves with complicated parameter solving procedures [15]. The domain switching induced rate-dependent hysteresis characteristic of piezoelectric ceramics has been deeply analyzed in material modeling research [16]. Phenomenological models abandon material microscopic analysis and characterize hysteresis via input-output relations, thus enjoying wider application. The Preisach model constructs complex hysteresis through weighted Relay operators with strong generalization, yet it demands massive experimental data for weight identification and suffers heavy computation burden [17]. The Prandtl-Ishlinskii (PI) model adopts continuous Stop operators with a concise structure, while it fails to depict asymmetric and rate-dependent hysteresis [18]. As an improved variant of the Preisach model, the KP model employs continuous operators to capture complex minor hysteresis loops with fewer units, balancing modeling precision and computational efficiency, making it a mainstream research focus [19]. Intelligent models build nonlinear mappings to reproduce hysteresis. Xu et al. [20] put forward a generalized regression neural network based on inverse Duhem operators, converting multi-valued hysteresis mappings into one-to-one relations to well characterize rate-dependent hysteresis.

The control strategies mainly fall into open-loop, closed-loop and compound control. Open-loop control based on inverse models owns simple structures yet weak anti-interference performance, whose compensation accuracy fully relies on model fitting quality. Model-free closed-loop control including PID and adaptive control features strong robustness, but its tracking speed and precision are constrained. Compound control combining feedback and feedback correction has become the dominant scheme. Lin et al. integrated the Preisach inverse model with PID regulation to cut maximum positioning error [21], and Zhu et al. constructed a dynamic PI compound controller that incorporates power amplifier dynamics to improve tracking precision for rate-dependent hysteresis [22]. Nevertheless, existing compound control approaches face obvious drawbacks: offline identified model parameters lose compensation accuracy under varying excitation frequency, amplitude and external disturbances, which creates urgent demand for adaptive control capable of online parameter updating.

Current micro-positioning stages have achieved great progress in multi-degree-of-freedom layout and large travel range. Nevertheless, hysteresis nonlinearity remains the core bottleneck restricting positioning precision. Among model-based control methods, the KP model stands out with balanced accuracy and low complexity. However, most existing KP control schemes only adopt offline parameter identification and lack adaptability to time-varying operating environments. Developing a well-designed piezoelectric flexure micro-positioning stage matched with KP-based adaptive control can realize accurate hysteresis modeling and real-time compensation, which bears important theoretical and engineering significance.

The KP hysteresis model and adaptive control are mostly studied separately in existing literature, with limited research integrating them to suppress piezoelectric hysteresis. The weight parameters of the KP model can be obtained through offline identification using pre-measured input-output data, yet its prediction accuracy degrades sharply once excitation frequency or ambient temperature changes, which requires repeated re-calibration. Traditional adaptive control can adjust controller parameters online but cannot directly compensate the multi-valued mapping of piezoelectric hysteresis. Combining the KP model with adaptive control offers a feasible way to update KP weights during real-time hysteresis compensation [23].

Motivated by this research gap, an adaptive inverse control is proposed with KP model for hysteresis compensation in piezoelectric flexure micro-positioning stage. A feedback branch is designed with displacement sensor to calculate the control error for adaptive updating. The parameters including KP weights are updated by proposed algorithm in real-time control, which do not need the pre-measured input-output data. It eliminates static pre-calibration and auxiliary dynamic correction links and effectively suppresses the rate-dependent hysteresis nonlinearity of piezoelectric flexure micro-positioning stage.

## 2. Design and Analysis of the Piezoelectric Flexure Micro-Positioning Stage System

### 2.1. Working Principle

**Figure 5:**
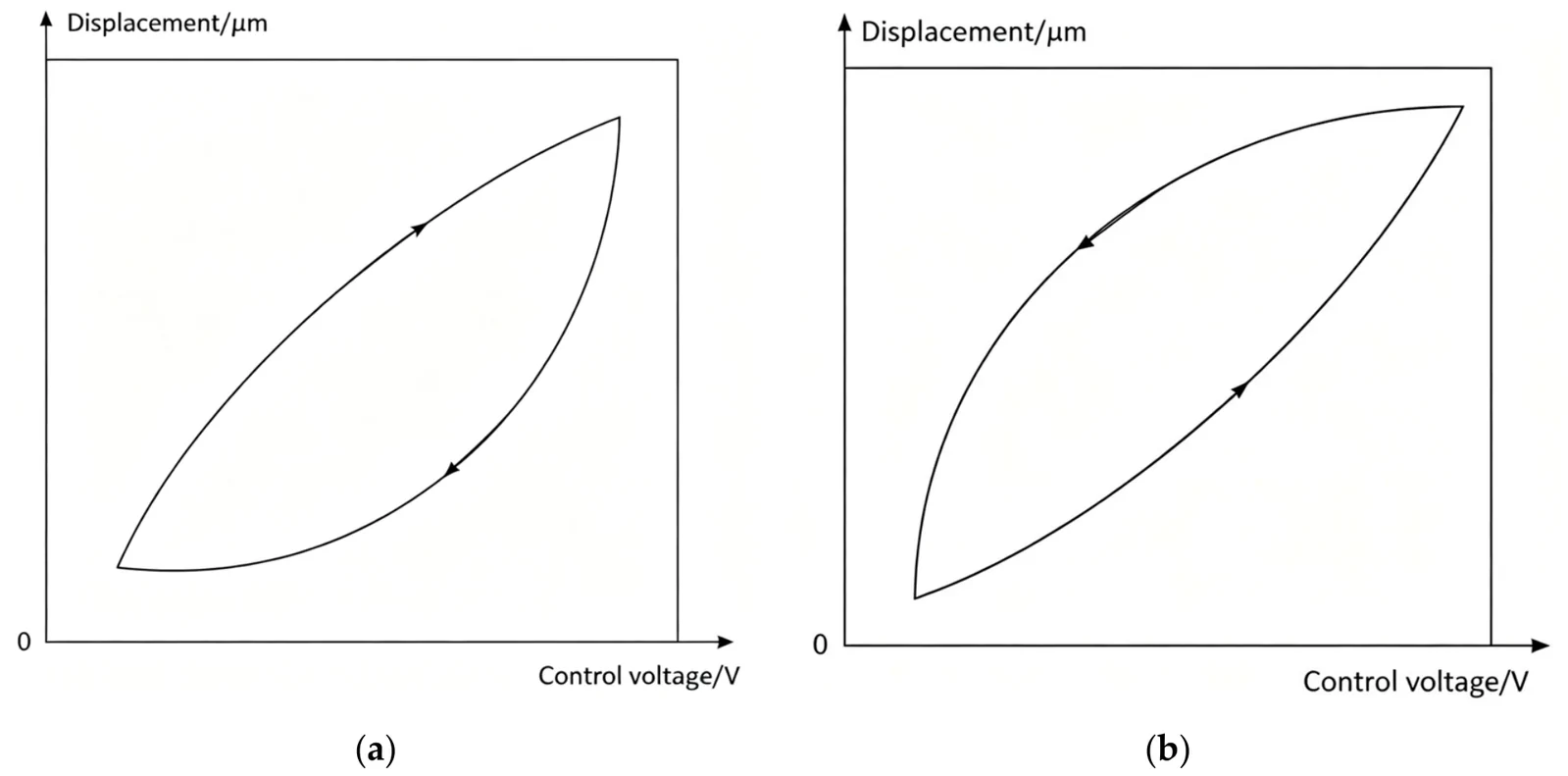
Hysteresis characteristic curves of piezoelectric stacks. (**a**) Low-frequency hysteresis loop; (**b**) Rate-dependent hysteresis loop.

**Figure 6:**
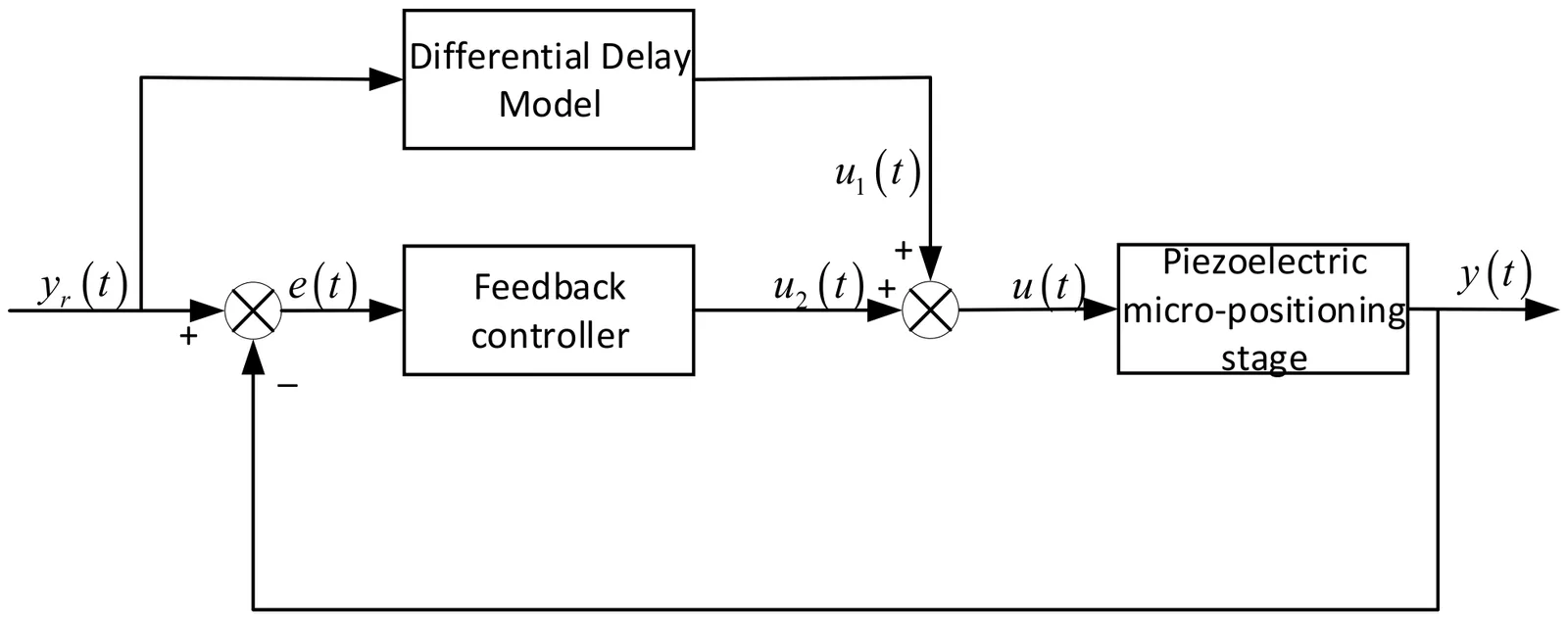
Block diagram of the drive control principle of the piezoelectric micro-positioning stage based on adaptive inverse control.

The piezoelectric flexure micro-positioning stage is an actuating mechanism with a small movement stroke, fast response speed and high positioning accuracy. It is a precise driving device for achieving micro-meter-level displacement positioning. Its working principle is that the trigger signal first transmits the start command to the control board. The upper computer sends positioning parameters to the control board via serial port. After receiving them, the control board generates a low-voltage control signal and simultaneously sends the system status back to the upper computer. Based on the KP hysteresis model, the adaptive inverse control algorithm embedded in the control board first receives the desired displacement of the motion stage as the input, calculates the corresponding pre-compensated driving voltage through the established KP inverse model, and dynamically adjusts the model parameters in real time according to the feedback displacement of the stage to offset the inherent hysteresis nonlinearity of the piezoelectric stack. The power supply provides the electrical energy required for the control board and the driving board to operate. The driving board amplifies the low-voltage control signal output by the control board into the high-voltage signal required by the piezoelectric stack. Subsequently, this high-voltage signal acts on the micro-positioning stage as the load device. The piezoelectric stack in the stage converts the electrical energy into its own mechanical micro-displacement through the inverse piezoelectric effect. The straight circular flexure hinge then transmits this micro-displacement directionally through elastic deformation, eliminating the friction and gap of traditional mechanical hinges. Finally, micro-displacement is amplified by the symmetrical two-stage displacement amplification mechanism. The amplified displacement is then transmitted to the motion stage to achieve high-precision positioning of tens to hundreds of micrometers. The voltage-displacement hysteresis characteristic curves of piezoelectric stacks are depicted in Figure 5, and the overall block diagram of the proposed adaptive inverse control system is shown in Figure 6.

### 2.2. Structural Design of Micro Positioning Stage

**Figure 7:**
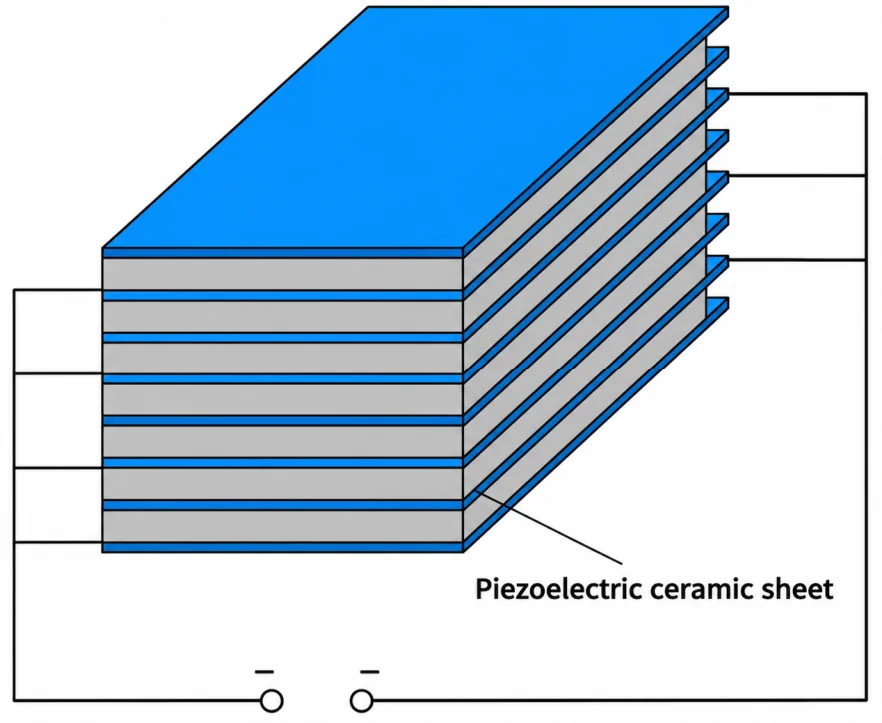
Piezoelectric stack actuator.

**Figure 8:**
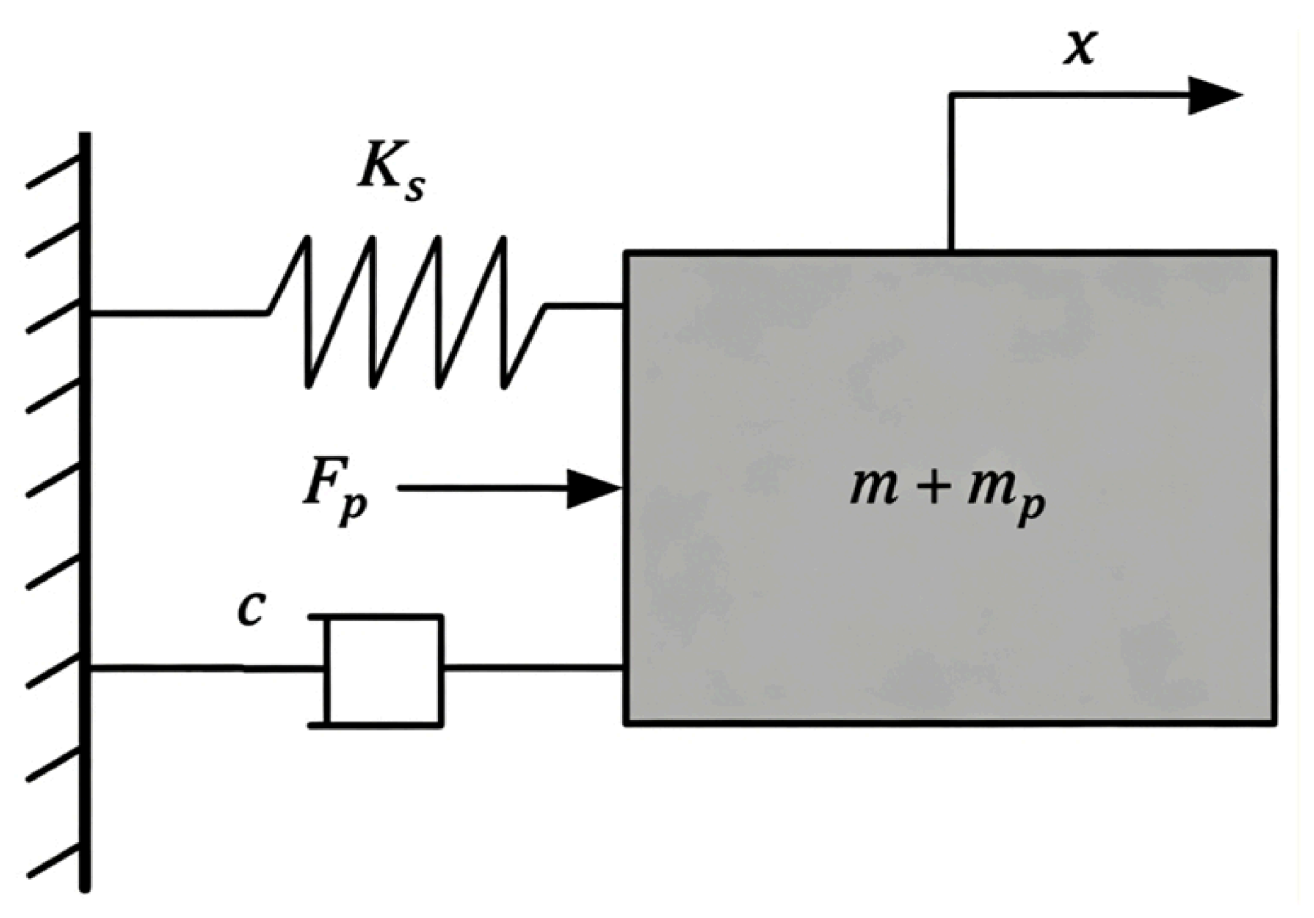
Piezoelectric stack simplified dynamic model.

As the core driving force of the stage, the piezoelectric stack, by virtue of the inverse piezoelectric effect, directly converts electrical energy into mechanical micro-displacements, providing the basic driving force for the subsequent displacement transmission and amplification. As shown in Figure 7 and Figure 8. Its dynamic characteristics can be approximated as a classic mass-spring-damper vibration system. The core formula is as follows:

Displacement and voltage relationship:

(1) $$\Delta x=n\cdot d\cdot v(t)+\alpha \cdot v{\left(t\right)}^{2}$$

Among them, Δx represents the output displacement of the piezoelectric stack, n represents the number of piezoelectric ceramic sheets inside the stack, d is the piezoelectric strain constant, v(t) is the externally applied voltage signal, α is the electro strictive coefficient.

Dynamic equation:

(2) $$m\ddot{x}+c\dot{x}+K_{s}x=F_{p}$$

where, m is the equivalent total mass of the positioning moving stage. $\ddot{x}$ is the acceleration of the stage output displacement. c is the equivalent damping coefficient of the flexure hinge mechanism. $\dot{x}$ is the velocity of the stage output displacement. $K_{s}$ is the overall equivalent stiffness of the flexible amplification structure. x is the actual output displacement of the micro-positioning stage. $F_{p}$ is the driving force output by the piezoelectric stack actuator.

**Figure 9:**
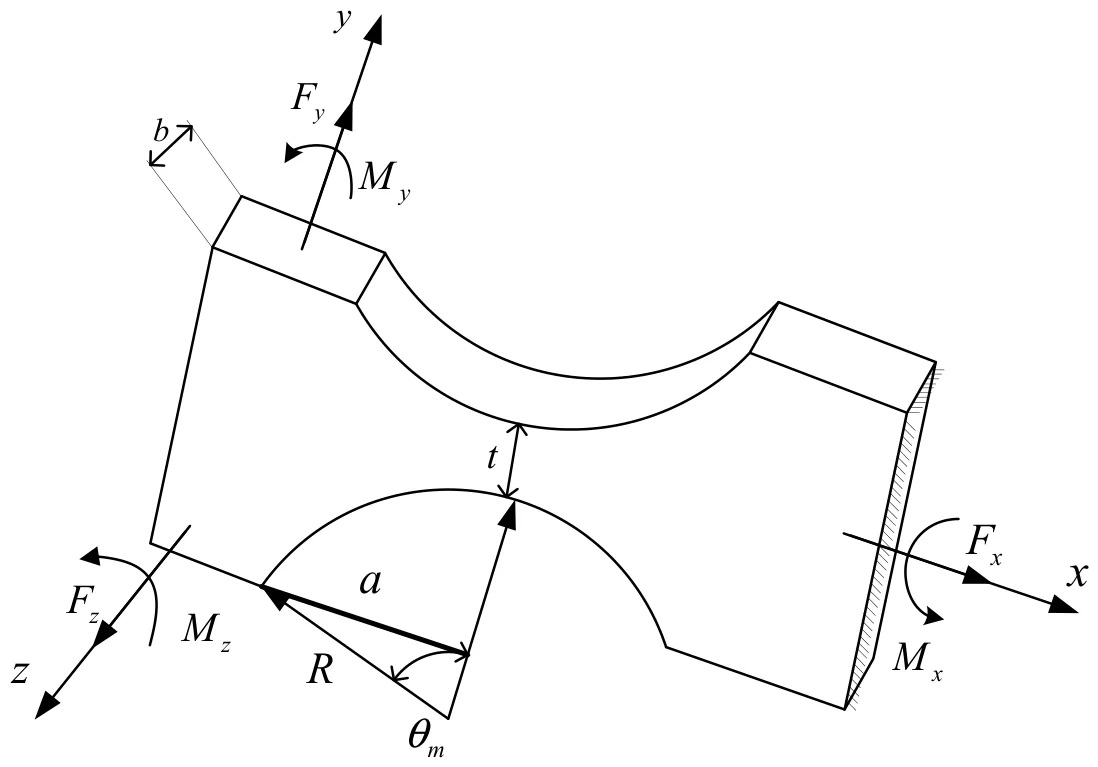
The geometric structure of the straight circular flexure hinge.

To ensure the accuracy and stability of displacement transmission, the stage adopts straight circular flexure hinges as the guiding mechanism. As shown in Figure 9. It achieves directional displacement transmission through the slight deformation of elastic materials, eliminating the friction and gap problems of traditional mechanical hinges, and effectively improving the positioning accuracy. Its core performance is characterized by flexibility expression. The core formula is as follows:

(3) $$\frac{1}{K_{z\theta }}=\frac{12R^{2}(1+\mu_{1})}{Ebt^{2}s^{2}}\cdot \frac{s^{2}+2s+2}{s+1}$$

Among them, $K_{z\theta }$ represents the angular stiffness along the *z*-axis, R is the radius of the hinge arc, t is the thickness of the hinge, b is the width of the hinge, E is the elastic modulus of the material, and $\mu_{1}$ is the Poisson’s ratio of the material.

**Figure 10:**
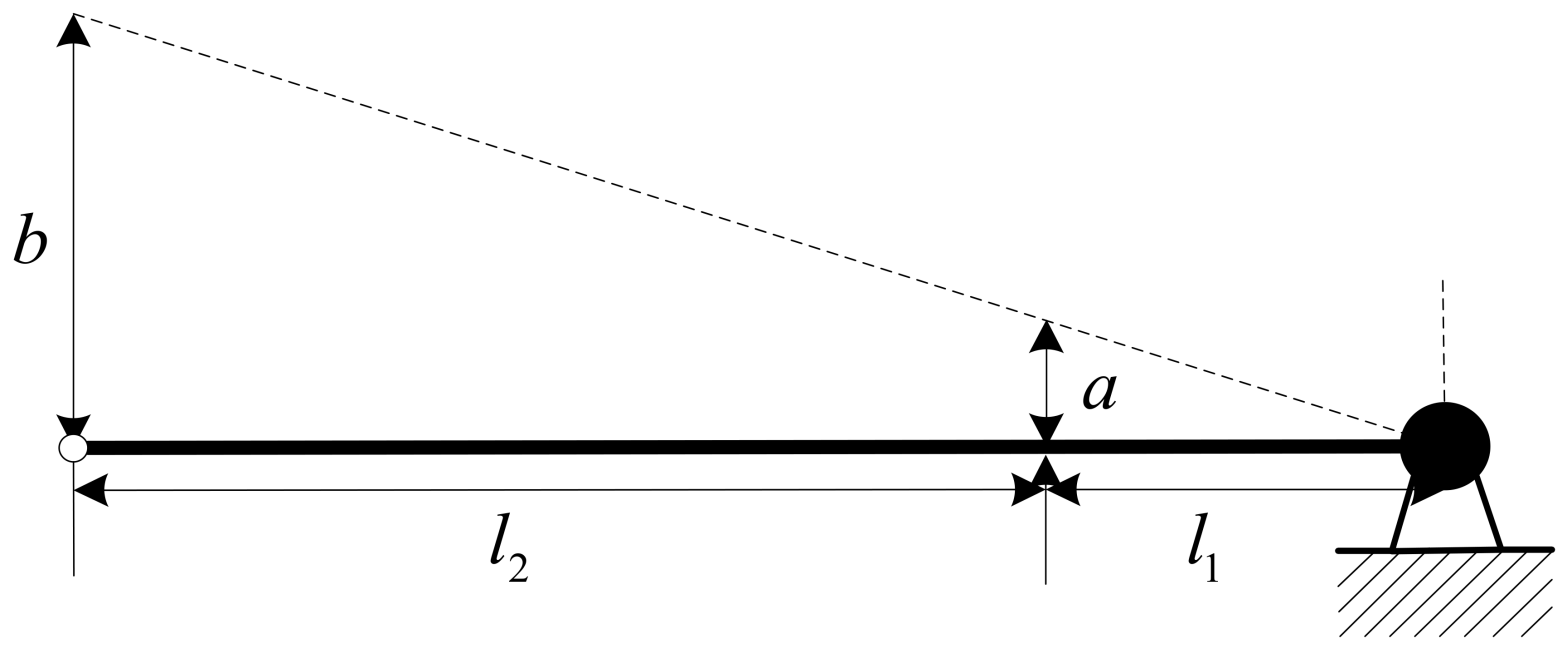
Primary leverage amplification mechanism.

Considering that the output displacement of the piezoelectric stack often fails to directly meet the requirements of practical applications, the stage is designed with a symmetrical two-stage displacement amplification mechanism. Through the force arm matching the two-stage levers, the displacement is progressively amplified. At the same time, the symmetrical structure is used to weaken the longitudinal coupling displacement error, ensuring the linear relationship between the input and output. The core formula is as follows:

The first-level amplification mechanism is shown in Figure 10. The right end is the fixed point, and its amplification factor $\lambda_{1}$ is:

(4) $$\lambda_{1}=\frac{l_{2}}{l_{1}}$$

Here, $l_{1}$ represents the length of the primary lever’s driving arm, and $l_{2}$ represents the length of the primary lever’s resisting arm.

The amplification factor $\lambda_{2}$ of the second-level amplification mechanism is:

(5) $$\lambda_{2}=\frac{l_{4}}{l_{3}}$$

Here, $l_{3}$ represents the length of the secondary lever’s power arm, and $l_{4}$ represents the length of the secondary lever’s resistance arm.

**Figure 11:**
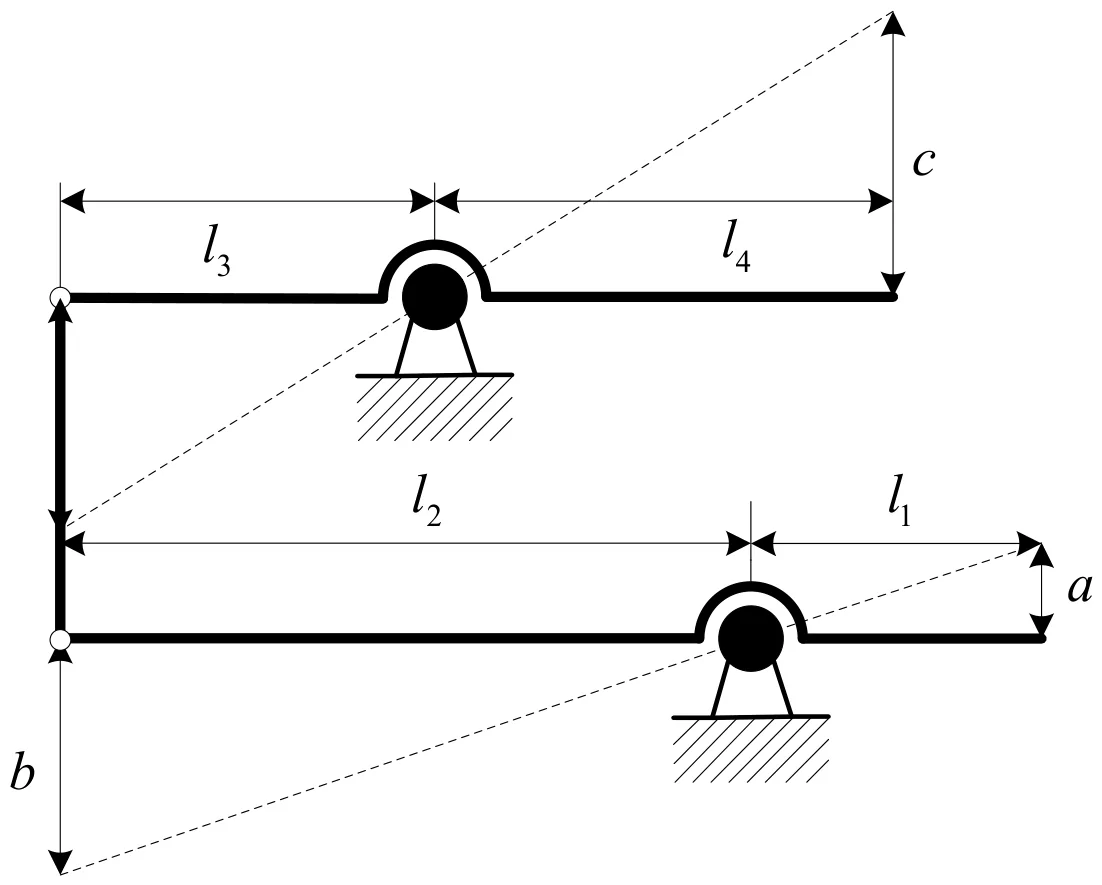
Diagram of the amplification principle of the secondary lever mechanism.

According to the principle of secondary leverage amplification (as shown in Figure 11), it can be deduced that the secondary leverage amplification factor λ is the product of the amplification coefficients of each level:

(6) $$\lambda =\lambda_{1}\cdot \lambda_{2}$$

**Figure 12:**
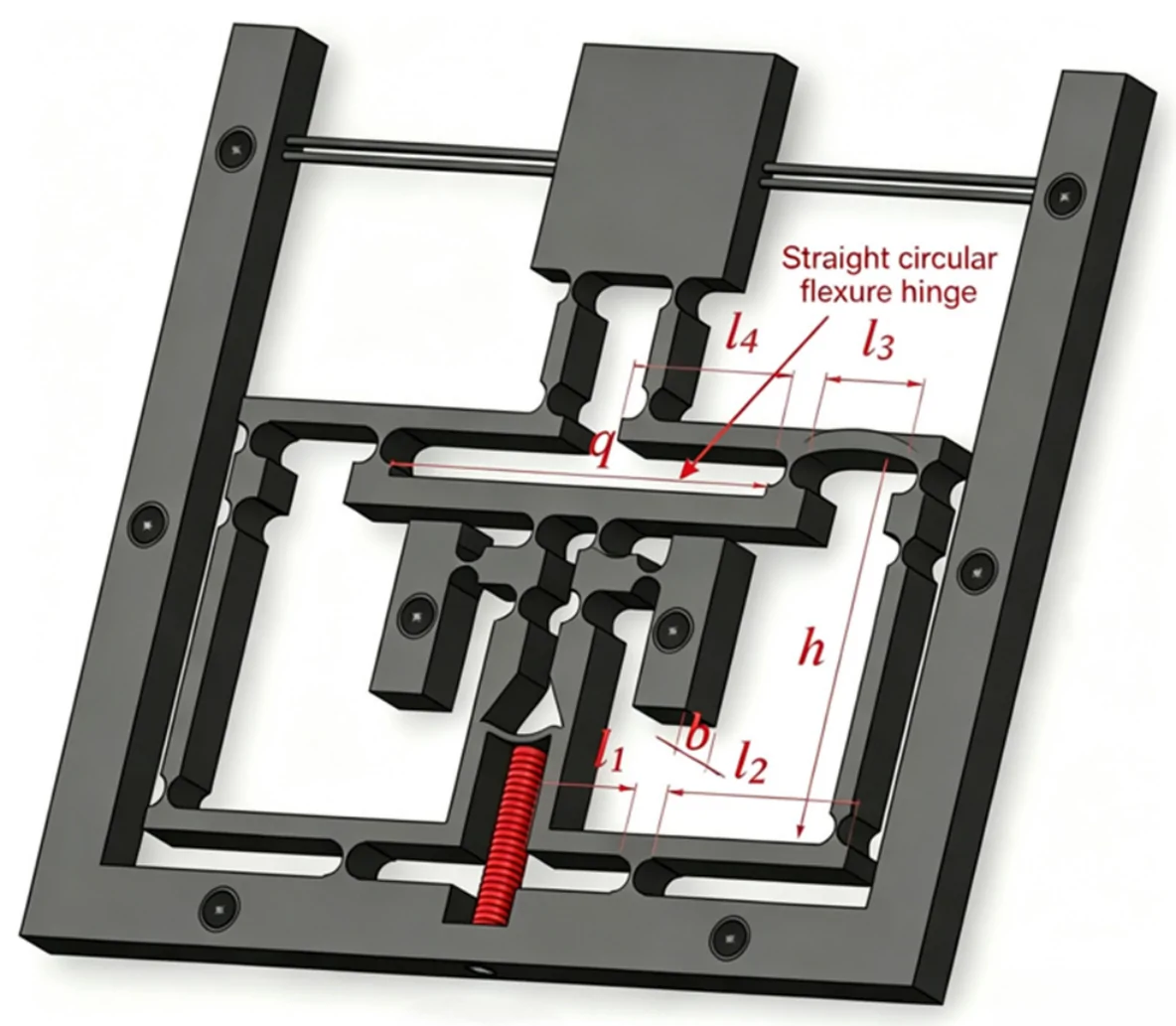
Micro-positioning stage structure diagram.

From Formula (6), it can be concluded that the secondary lever amplification factor λ is directly proportional to $l_{2}$ and $l_{4}$, and inversely proportional to $l_{1}$ and $l_{3}$. The lengths of $l_{2}$ and $l_{4}$ can be adjusted to meet the requirements of the secondary lever amplification factor. The structural diagram of the designed micro-positioning stage is shown in Figure 12.

### 2.3. Finite Element Simulation Analysis

**Figure 13:**
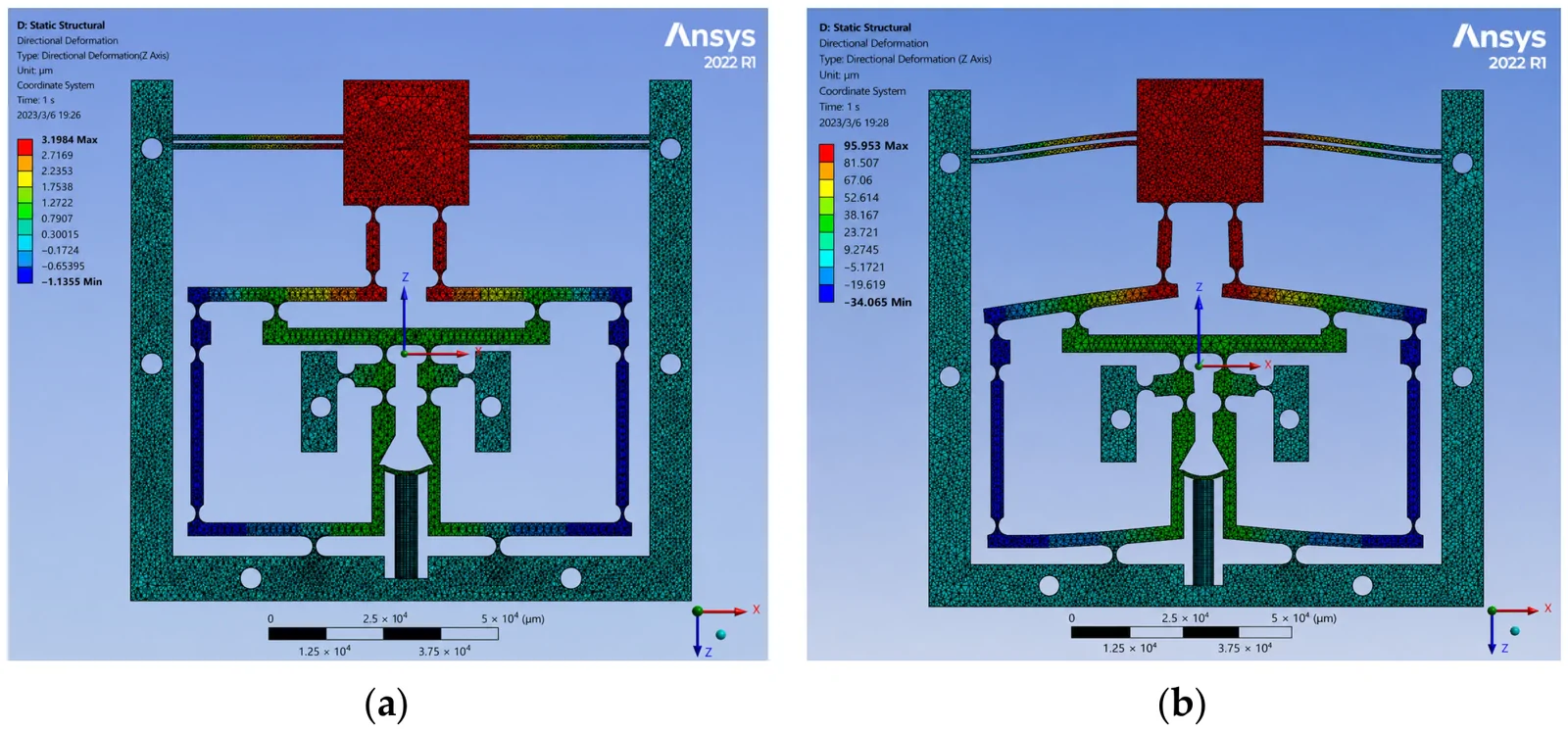
Displacement output simulation diagram. (**a**) 1 μm input; (**b**) 30 μm input.

Different amplitude displacement loads were applied at the input end of the piezoelectric stack, and the output displacement response of the stage was solved. The simulation results showed that there was a good linear relationship between the input and output displacements of the stage. A stable displacement amplification effect was achieved through a secondary lever amplification mechanism, with the maximum output displacement reaching 95.95 μm, which met the intermediate travel requirements of the design. The quantitative correspondence between input displacement and simulated output displacement at multiple loading points is summarized in Table 1. The simulation results of the directional deformation under typical input displacements are shown in Figure 13. Figure 13a shows the displacement distribution when the input displacement is at a small value, and Figure 13b shows the simulation cloud map of the maximum output displacement corresponding to the maximum input displacement.

Multiple quantitative evaluation metrics including maximum output stroke and linearity error are extracted from the displacement simulation data.

**Figure 14:**
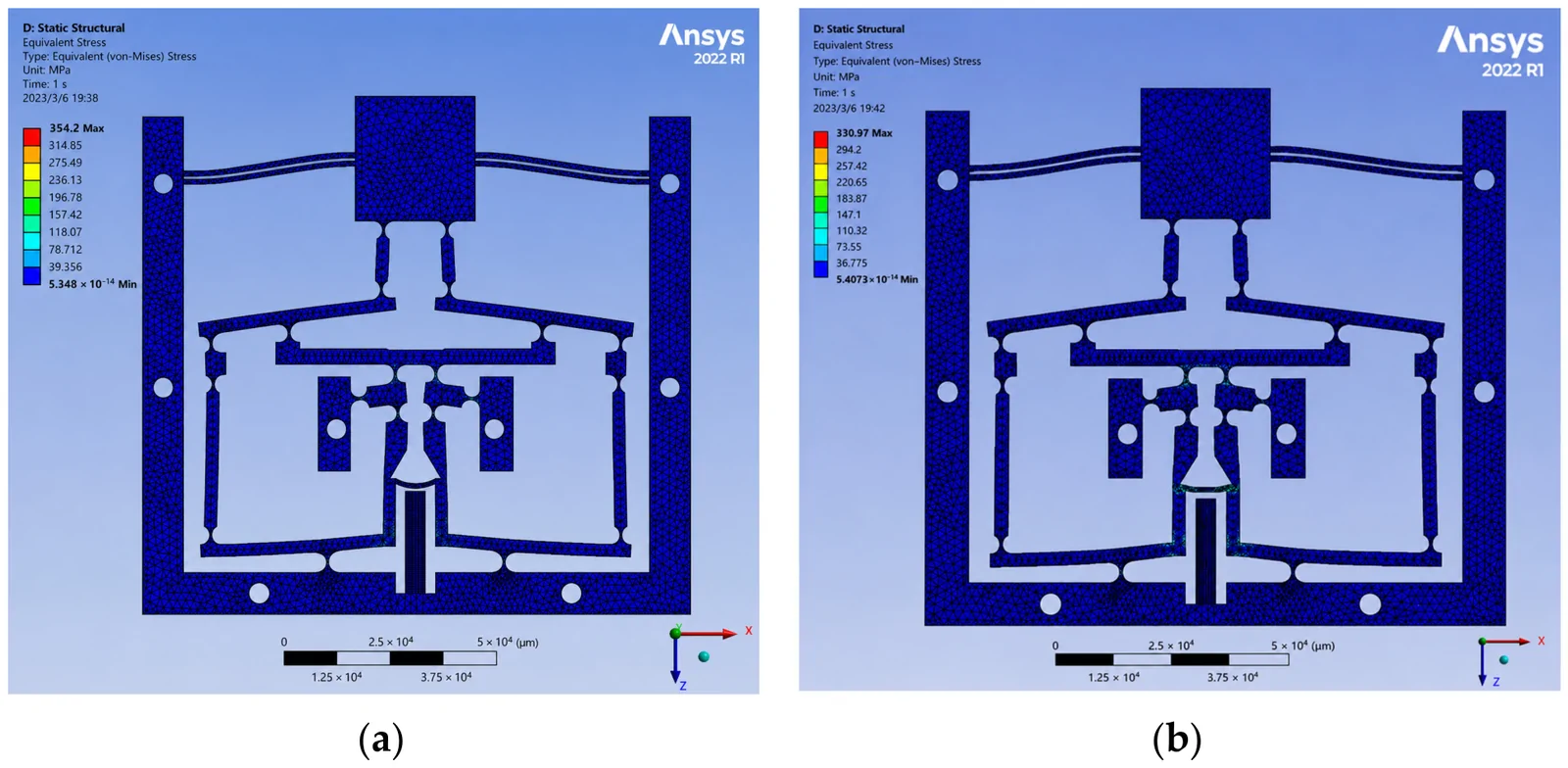
Equivalent stress simulation diagram. (**a**) Displacement condition; (**b**) Stress conditions.

To verify the strength and safety of the stage structure, stress analysis was conducted for the maximum input displacement condition and the rated driving force condition. Simulation results show maximum equivalent stress concentrates on arc notches of straight circular flexure hinges and rotation centers of lever amplifiers. The maximum stresses under the two conditions are 354.2 MPa and 330.97 MPa respectively, which are both far below the yield strength of P20 tool steel. The structural strength meets the design requirements. The stress simulation cloud map is shown in Figure 14, where Figure 14a shows the stress distribution under the displacement load and Figure 14b shows the stress distribution under the force load.

**Figure 15:**
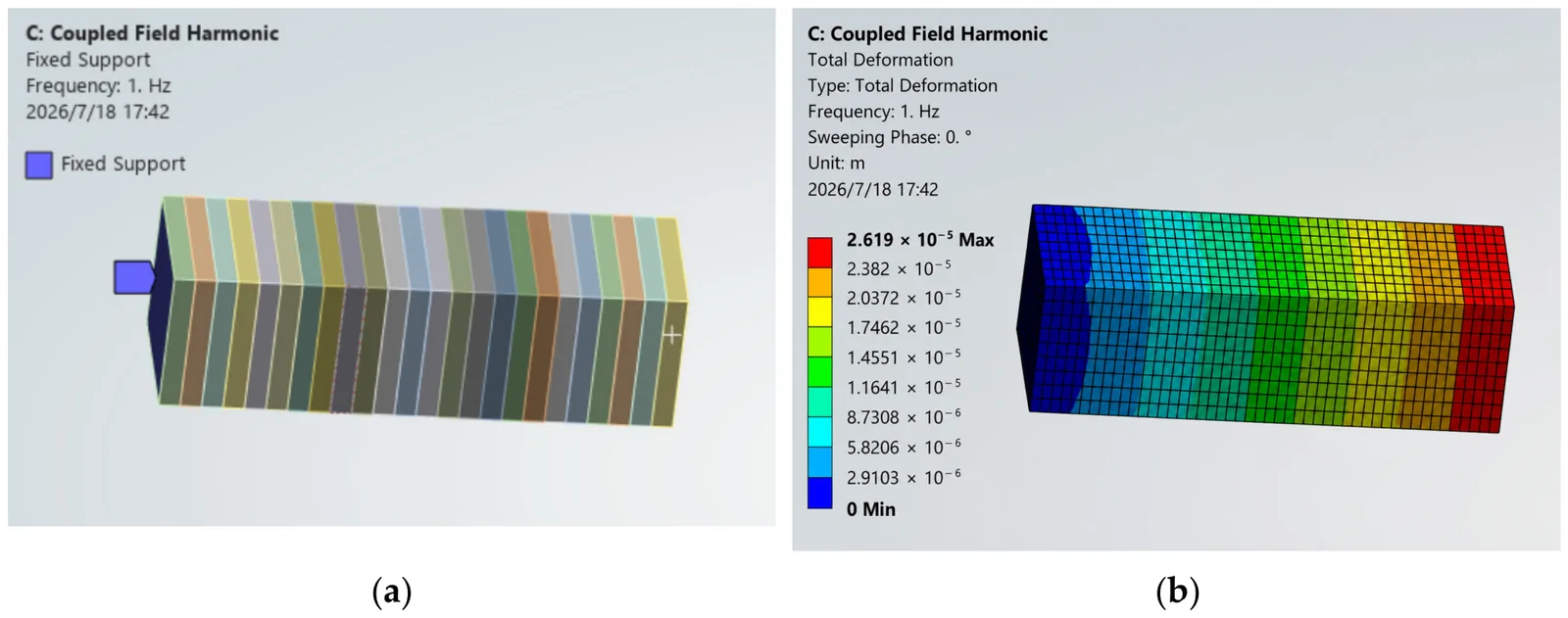
Schematic diagram and coupled harmonic field simulation of piezoelectric stack actuator. (**a**) Diagram of piezoelectric stack actuator; (**b**) Simulation of piezoelectric actuator.

The piezoelectric actuator equipped in the proposed structure is a kind stack actuator which is made of 24 piezoelectric wafers. The structure model of piezoelectric actuator was established in SolidWorks software, and loaded to ANSYS software. An ending surface of first piezoelectric wafer was set to be fixed support as shown in Figure 15a, and another ending surface of last surface is preloaded. The contact surfaces of piezoelectric wafers are bonded, and voltage applied on every piezoelectric wafer is the same. After choosing the material of PZTR-5H, the result is solved in coupled field harmonic module. As shown in Figure 15b, the maximum elongation is 26.19 μm, which is suitable for designed micro-positioning stage.

**Figure 16:**
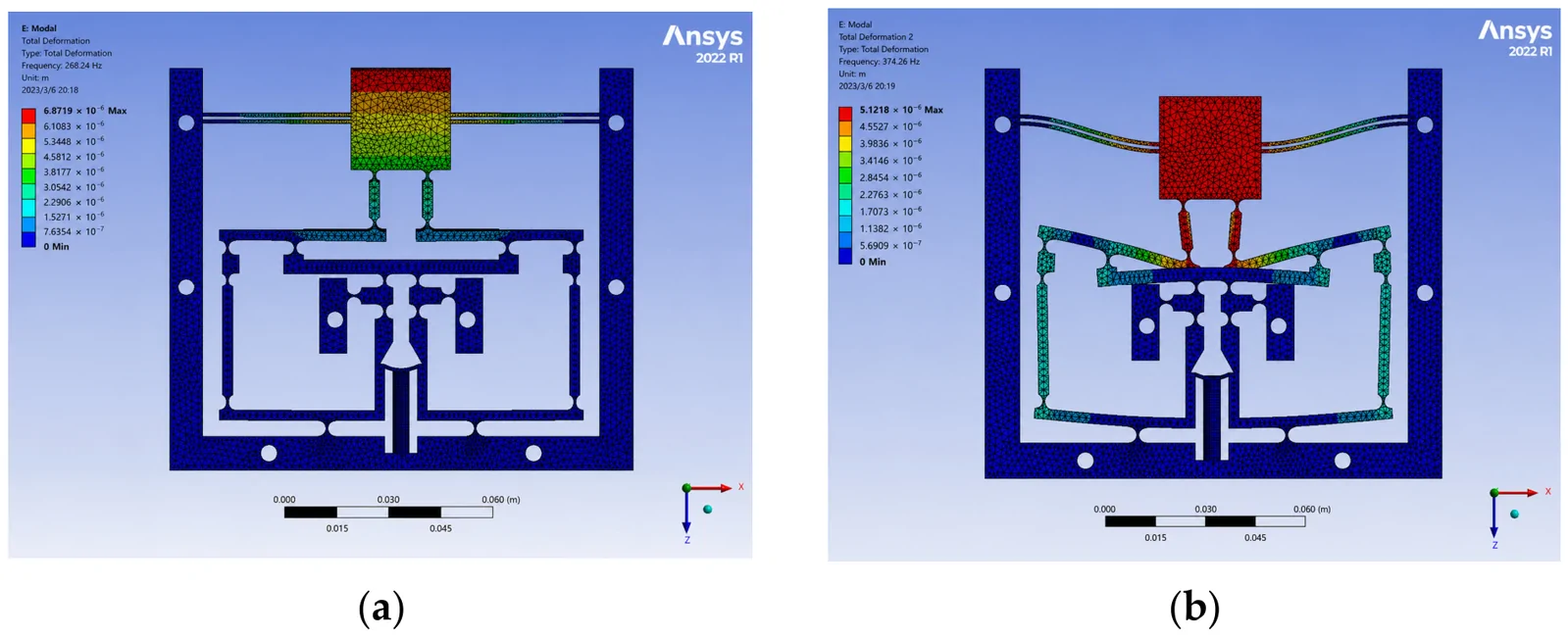

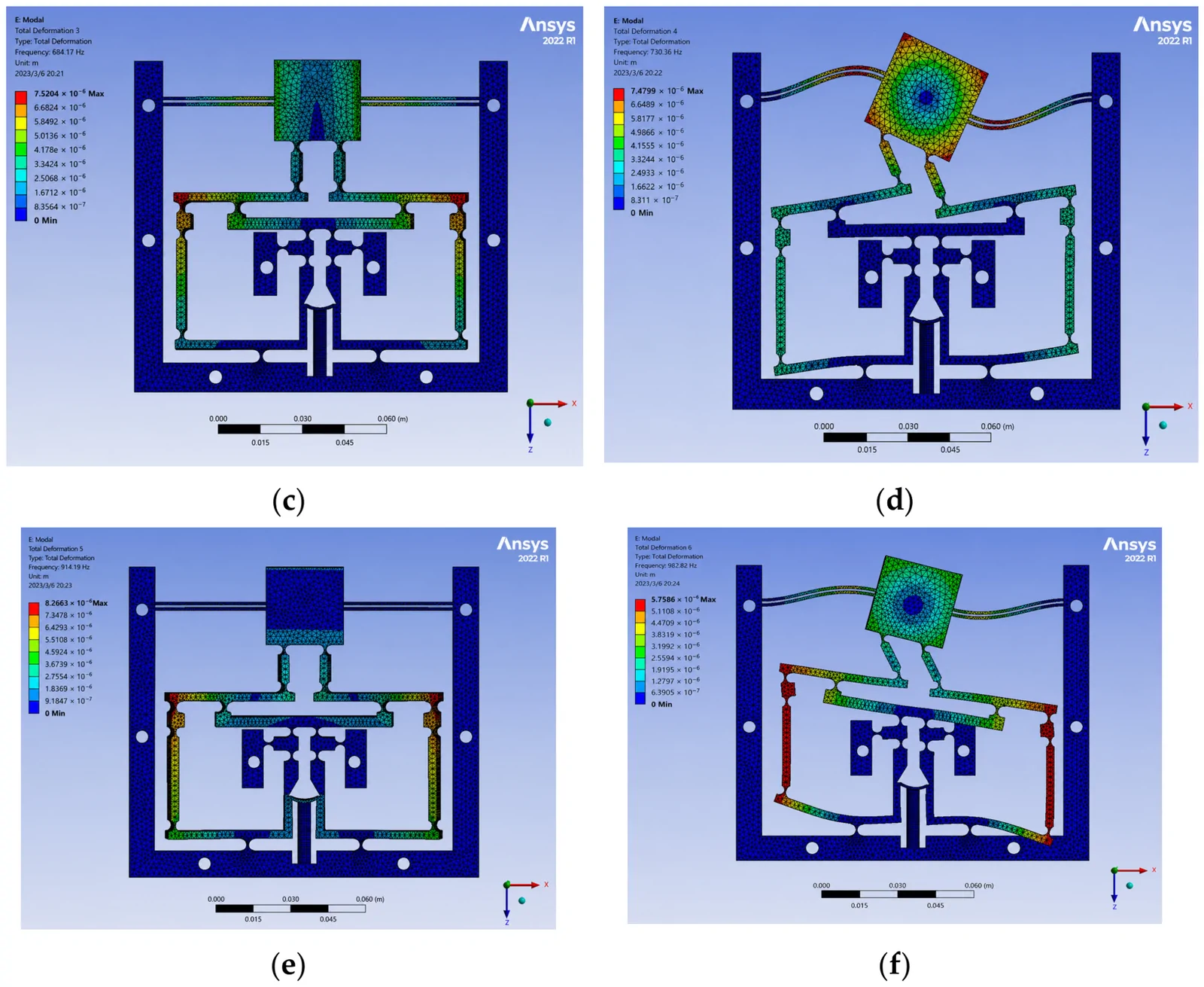
The first six natural vibration modes of the micro-positioning stage. (**a**) First-order modal shape; (**b**) Second-order modal shape; (**c**) Third-order modal vibration mode; (**d**) Fourth-order modal vibration mode; (**e**) Fifth-order modal vibration mode; (**f**) Sixth-order modal vibration mode.

To validate the dynamic rationality and avoid resonance failure of the designed single-degree-of-freedom piezoelectric micro-positioning stage, modal analysis is carried out via ANSYS Workbench to extract the first six natural vibration modes and corresponding natural frequencies of the whole compliant structure. The first six modal shapes are displayed in Figure 16, and the detailed natural frequency values of each order are summarized in Table 2.

## 3. KP Hysteresis Modeling and Adaptive Inverse Control Design

### 3.1. Classical KP Hysteresis Model

The core idea of the KP model is to precisely describe the complex hysteresis behavior of piezoelectric actuators by weighing and superimposing several KP operators with hysteresis characteristics. Its general mathematical expression is:

(7) $$u(t)=\iint_{P}\mu (s_{1},s_{2})K_{s_{1},s_{2}}[w(t),K_{\upsilon -1}]ds_{1}ds_{2}$$

Among them, u(t) represents the output of the model, $K_{s_{1},s_{2}}[w(t),K_{\upsilon -1}]$ represents the KP operator, $\mu (s_{1},s_{2})$ is the weight function, and P is the Preisach integral plane:

(8) $$P=\left\{p(s_{1},s_{2})\in R\times R:-u^{-}\leq s_{1}\leq s_{2}\leq u^{+}-a\right\}$$

Among them, $u^{+}$ represents the positive hysteresis input saturation value, $u^{-}$ represents the negative hysteresis input saturation value, and *a* is the increasing constant of the KP operator. By defining the parameter P, the output equation value of each KP operator can be determined.

**Figure 17:**
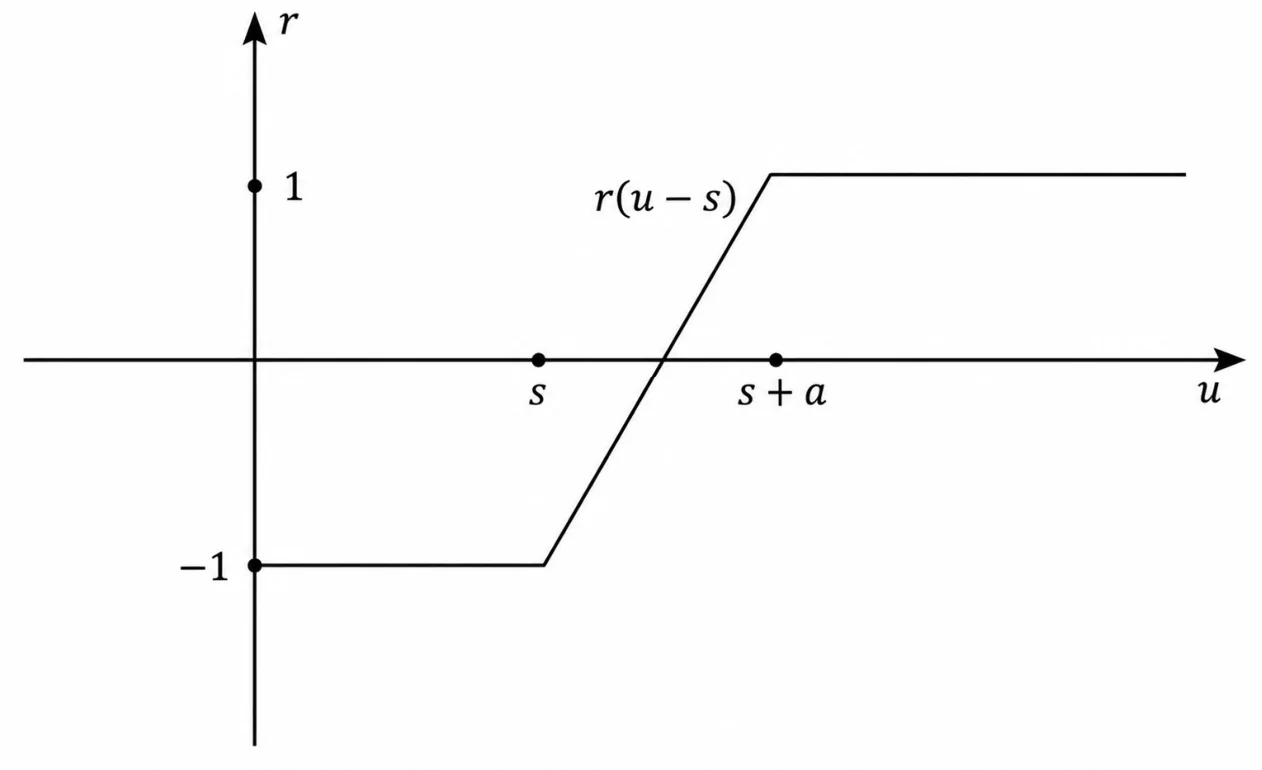
The ascending or descending section of the KP operator.

**Figure 18:**
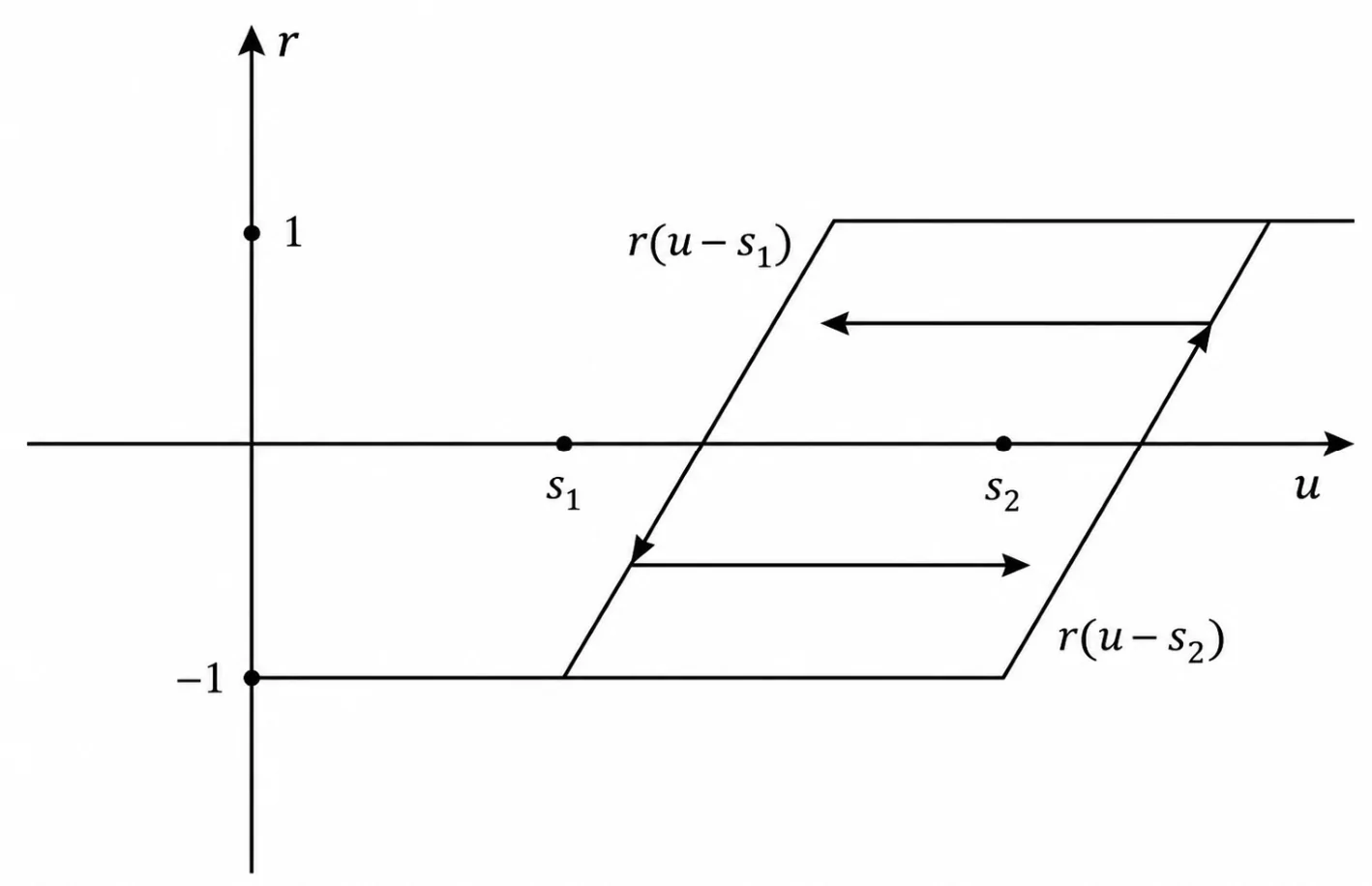
KP hysteresis operator.

The range of value of the KP operator is [−1, 1]. Its output characteristics are determined by the input signal u(t), the previous output value, and the turning point parameters $s_{1}$ and $s_{2}$. It can be divided into two core stages: the rising stage and the falling stage. The segmented characteristics are shown in Figure 17, and the complete KP hysteresis operator curve is shown in Figure 18.

Based on the above characteristics, the function expression of the KP hysteresis operator can be defined as:

(9) $$\left[K(w(t),K_{\upsilon -1})\right]\left(t\right)=\left\{\begin{array}{l} \max\left\{K_{\upsilon -1},r(w(t)-s_{2})\right\},dw\geq 0\\ \min\left\{K_{\upsilon -1},r(w(t)-s_{1})\right\},dw\leq 0 \end{array}\right.$$

Among them, $K_{\upsilon }$ is mainly influenced by the historical extreme values of the KP operator and changes along with the variation of the delayed input. Its expression can be described as:

(10) $$K_{\upsilon }=\left\{\begin{array}{l} K(u,K_{\upsilon -1})\left(t_{k}\right),k=2,\ldots ,j\\ K_{0}=\xi ,\upsilon =1,\xi \in \left\{-1,1\right\} \end{array}\right.$$

In this formula, $K_{\upsilon }$ represents the output state of the elementary hysteresis operator at the *v*-th sampling instant, $K(u,K_{\upsilon -1})\left(t_{k}\right)$ is the updated operator state calculated by the current input u and the previous state $K_{\upsilon -1}$ at discrete time $t_{k}$ for subsequent sampling instants, $K_{0}$ denotes the initial state of the elementary hysteresis operator, and ξ is the binary initial-state parameter chosen from the given set of two possible values when at the initial sampling instant.

### 3.2. Discretization of the Preisach Integral Plane

To reduce the computational complexity of the model and facilitate the subsequent engineering implementation, it is necessary to discretize the continuous Presiach integral plane. The architecture of KP model is not modified, the computational cost of KP model should be not changed. The integral plane is uniformly divided into *L* × *L* grids, and the number of discrete KP operators can be calculated by the following formula:

(11) $$N_{KP}=\frac{\left(L+1)(L+2\right)}{2}$$

**Figure 19:**
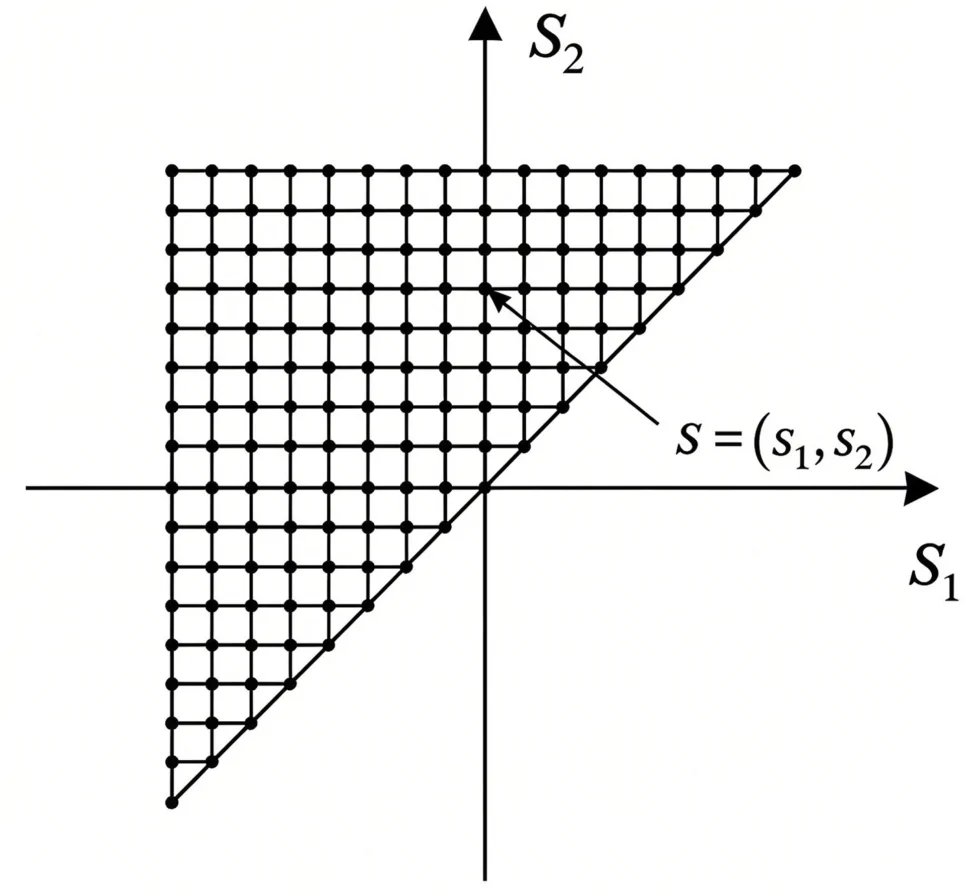
Discretized presiach integral plane.

The discretized Presiach integral plane is shown in Figure 19. Currently, the KP model is transformed from the continuous integral form to the weighted sum form of a finite number of KP operators, and its discretization expression is:

(12) $$R(t)=\sum_{i=1}^{N_{KP}}\mu_{i}K_{i}[u(t)]$$

**Figure 20:**
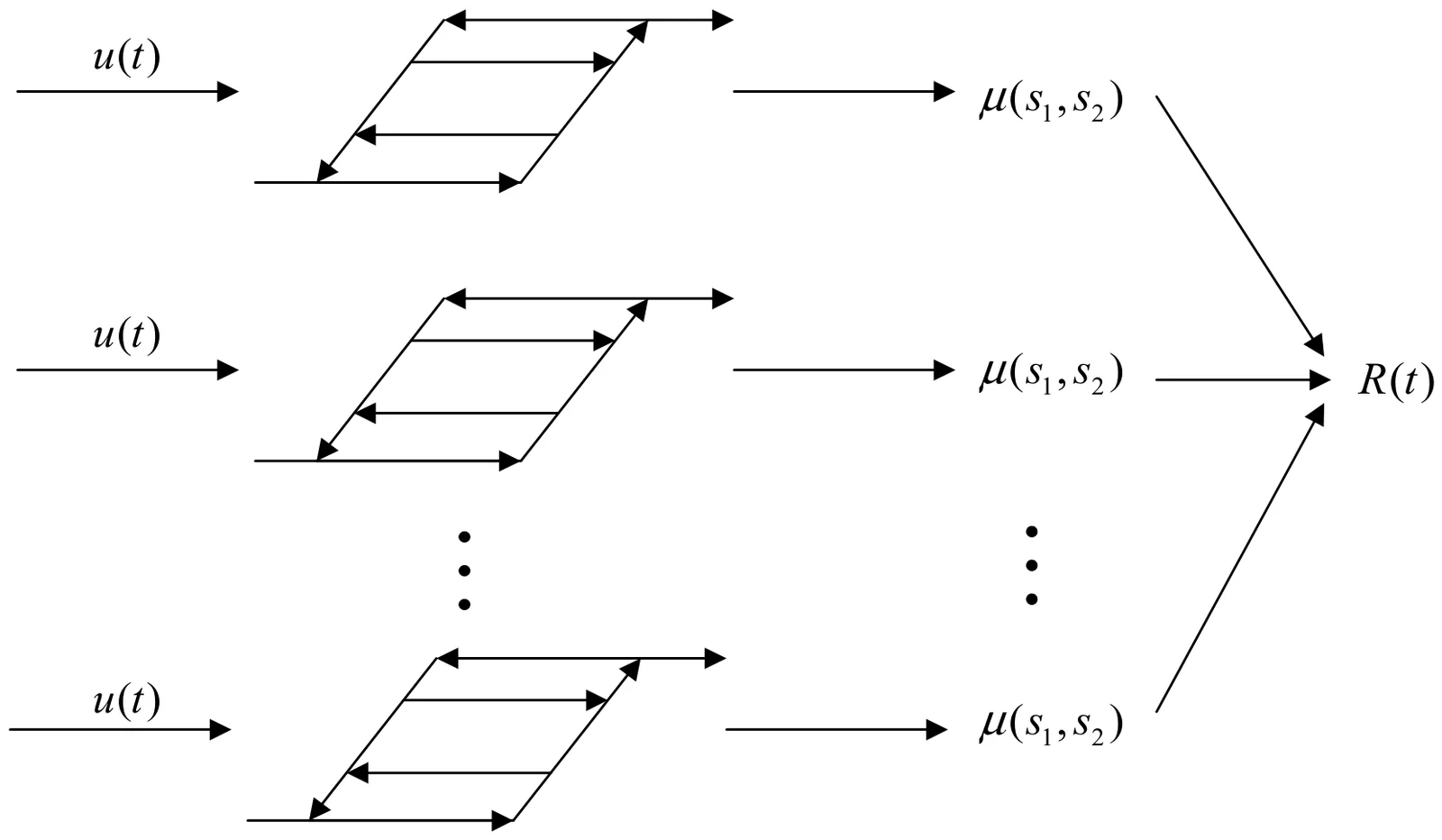
KP model composition diagram.

Among them, $\mu_{i}$ represents the weight of the *i*-th KP operator, and it is the output of the *i*-th KP operator after discretization. The composition logic of the KP model is shown in Figure 20. By superimposing KP operators with different weights, the overall hysteresis characteristic of the piezoelectric actuator can be reproduced.

The Preisach plane is the core theoretical framework of the model and the basis of the KP model. For a basic Relay operator, the total lagging output is the weighted superposition of all operator outputs. For the KP model, the continuous Preisach plane is discretized into an *L* × *L* grid, where each grid intersection represents a discrete threshold pair. This discrete grid converts the continuous double integration of the Preisach model into a discrete weighted sum, laying the foundation for modeling the KP operator. At the same time, it overcomes the discontinuity of the Relay operator in the continuous KP operator. The discretized Preisach plane provides a clear coordinate system for the KP operator, facilitating subsequent weighted superposition and parameter identification.

**Figure 21:**
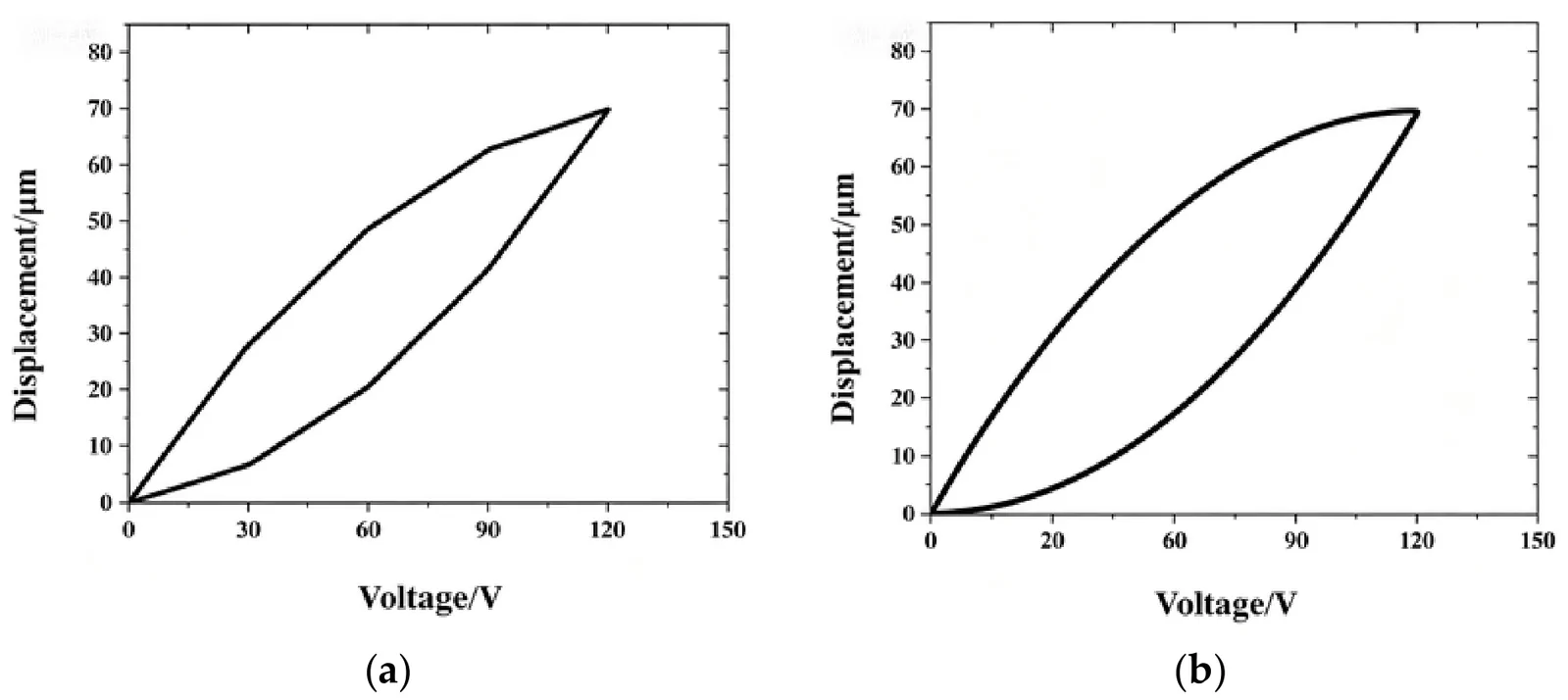
Simulation diagrams of KP models with different numbers of operators. (**a**) The KP model composed of 6 KP operators; (**b**) The KP model composed of 5050 KP operators.

As shown in Figure 21, the KP models were simulated separately by the software for the operation of 6 KP operators and 5050 KP operators. Each KP operator can only describe the hysteresis process in the form of a straight line, and the complete hysteresis curve is formed by the linear fitting of multiple KP operators. The results show that as the number of operators increases, the fitting effect becomes more obvious, and the model is more accurate in describing the hysteresis characteristics. Therefore, the smaller the model error is, the better the hysteresis compensation ability is, but the parameter identification calculation volume will increase.

### 3.3. Design of Adaptive Inverse Control Scheme

**Figure 22:**
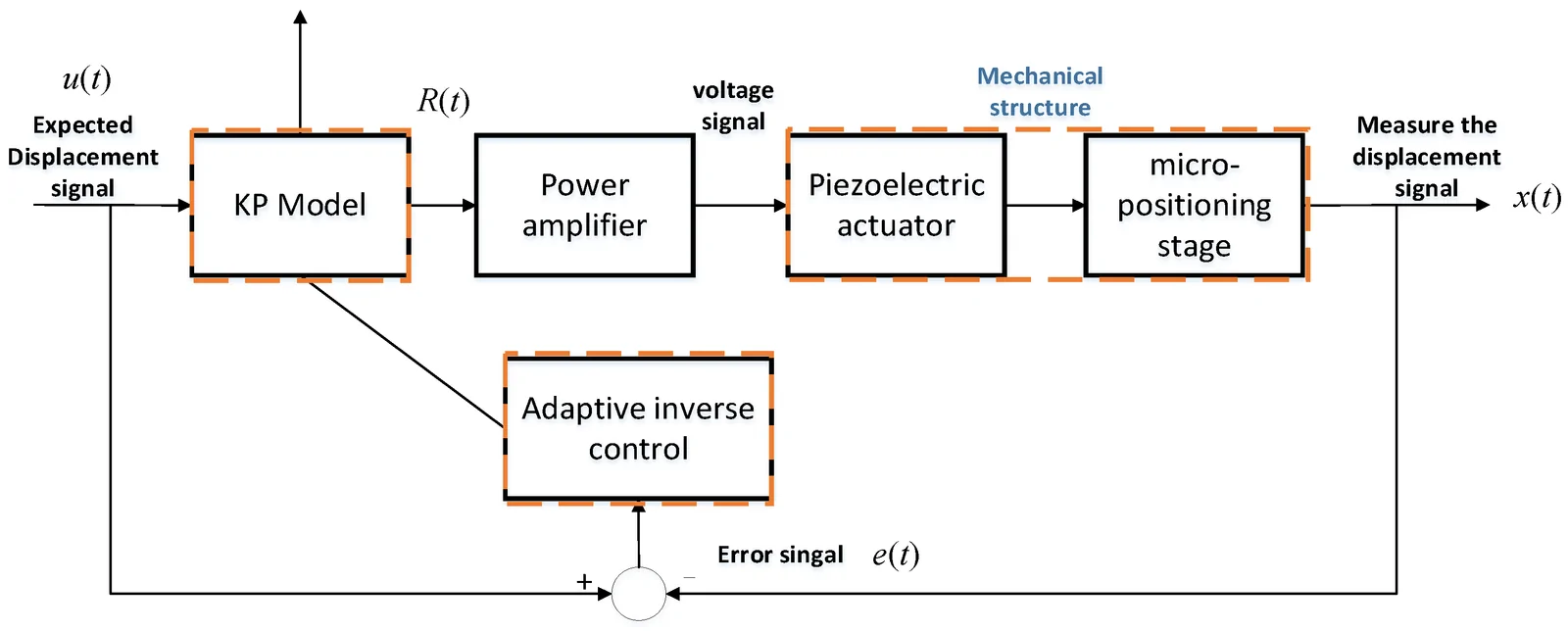
Adaptive inverse control system block diagram based on KP model.

The classic parameter identification method of KP model is a kind of off-line method which needs to measure the input and output data before identification [23]. When the model parameters identification is performing, the control process has to be suspended. The adaptive inverse control is proposed to perform the model parameters updating and hysteresis compensation in every sampling loop. The block diagram of proposed adaptive inverse control with KP model is shown in Figure 22, and the control error is defined as:

(13) $$e\left(t\right)\;=\;u_{1}\left(t\right)\;-\;x_{m}\left(t\right)$$

where $u_{1}\left(t\right)$ is the expected displacement and model input, $x_{m}(t)$ is the measured displacement. If the KP model is precise enough, the error e(t) should be zero. Theoretically, the error for model parameter identification should be defined with model error, but it is difficult to obtain the model error in real-time control. In instead of model error, the control error of Equation (13) can be achieved by displacement sensor and applied in the model parameters updating instead of model error [23,24]. Then the optimization objective is defined to minimize control error between expected displacement and measured displacement.

(14) $$\min J=\min\frac{e^{2}(t)}{2}=\min\frac{1}{2}{\left[u\left(t\right)-x_{m}\left(t\right)\right]}^{2}$$

Substitute Equation (12) into Equation (14), the discrete time form of its derivative to weight parameter $\mu_{i}(kT)$ is:

(15) $$\frac{\partial J}{\partial \mu_{i}\left(kT\right)}=-\frac{\partial x_{m}\left(kT\right)}{\partial \mu_{i}\left(kT\right)}=-\eta_{1}\;\frac{\partial R\left(kT\right)}{\partial \mu_{i}\left(kT\right)}=-\eta_{1}\;K_{i}[u(kT)]\;,k=0,1,2,3,\cdots$$

where T is the sampling period and k indicates the time series. The variable $\eta_{1}=\frac{\partial x_{m}(t)}{\partial R(kT)}$ depends on the inherit property of piezoelectric actuator, and its value is difficult to be measured. In practice engineering, its approximate value is usually obtained by trial and error. Then the updating of weight parameter is:

(16) $$\Delta \mu_{i}\left(kT\right)=\gamma \mu_{i}\left(kT-T\right)+\left(1-\gamma \right)\eta_{2}\eta_{1}K_{i}[u_{i}(kT-T)]$$

where $\eta_{2}$ is the learning rate of adaptive control, γ is momentum coefficient which can maintain stability with a larger learning rate. In control design, the parameter $\eta_{1}$ and $\eta_{2}$ can be considered as a single variable, then the updating of weight parameter $\mu_{i}(kT)$ is expressed as:

(17) $$\mu_{i}\left(kT\right)=\mu_{i}\left(kT\right)+\gamma \mu_{i}\left(kT-T\right)+\left(1-\gamma \right)\eta K_{i}[u_{i}(kT-T)]$$

where $\eta =\eta_{1}\eta_{2}$ mean a general learning rate. both η and γ are obtained by trial and error in real-time control experiment.

The stability of proposed adaptive control system has been discussed with consideration of hysteresis nonlinearity, then Equation (2) can be expressed as:

(18) $$m\ddot{x}+c\dot{x}+kx+f_{h}(x,\dot{x})=u$$

where $f_{h}$ indicates the hysteresis nonlinearity, which satisfy the boundary condition $\left|f_{h}\right|\leq \rho_{1}\left|x\right|+\rho_{2}\left|\dot{x}\right|$. Define the state variables $x_{1}=x$ and $x_{2}=\dot{x}$, then the Equation (18) of system can be written in state space form.

(19) $$\begin{array}{l} {\dot{x}}_{1}=x_{2}\\ {\dot{x}}_{2}=\frac{1}{m}\left(-kx_{1}-cx_{2}-f_{h}+u\right) \end{array}$$

Choose the original point $\left(x_{1}=0,x_{2}=0\right)$ as the expected equilibrium point, a positive definite quadratic form is selected as the basic framework function.

(20) $$W_{0}(x)=\frac{1}{2}mx_{2}^{2}+\frac{1}{2}kx_{1}^{2}$$

Take the multi-valued hysteresis into Equation (20), an adaptive parameter estimation term is introduced, and a complete composite Lyapunov function is constructed:

(21) $$W(x,\;{\tilde{\rho }}_{1},\;{\tilde{\rho }}_{2})=W_{0}(x)+\frac{1}{2q_{1}}{\tilde{\rho }}_{1}^{2}+\frac{1}{2q_{2}}{\tilde{\rho }}_{2}^{2}$$

where ${\tilde{\rho }}_{1}={\hat{\rho }}_{1}-\rho_{1}$ and ${\tilde{\rho }}_{2}={\hat{\rho }}_{2}-\rho_{2}$ are parameter estimation errors, $q_{1}$ and $q_{2}$ are adaptive gains which ensures the function is globally positive definite and radially unbounded. Substitute the Equation (19) into state space Equation (20).

(22) $$\dot{W}=mx_{2}{\dot{x}}_{2}+kx_{1}x_{2}+\frac{{\tilde{\rho }}_{1}{\dot{\hat{\rho }}}_{1}}{q_{1}}+\frac{{\tilde{\rho }}_{2}{\dot{\hat{\rho }}}_{2}}{q_{2}}$$

Choose the control input and adaptive parameter as:

(23) $$\begin{array}{l} u=-k_{1}x_{2}-{\hat{\rho }}_{1}\left|x_{1}\right|\mathrm{sgn}\left(x_{2}\right)-{\hat{\rho }}_{2}\left|x_{2}\right|\mathrm{sgn}\left(x_{2}\right)\\ {\dot{\hat{\rho }}}_{1}=q_{1}\left|x_{1}\right|\left|x_{2}\right|\\ {\dot{\hat{\rho }}}_{2}=q_{2}x_{2}^{2} \end{array}$$

It can be found that equation (22) is no greater than zero,

(24) $$\dot{W}\leq -cx_{2}^{2}-k_{1}x_{2}^{2}\leq 0$$

which means the system of Equation (18) eventually converge to the origin, achieve global asymptotic stability.

## 4. Experimental Setup and Performance Validation

### 4.1. Hardware Configuration of the Test System

**Figure 23:**
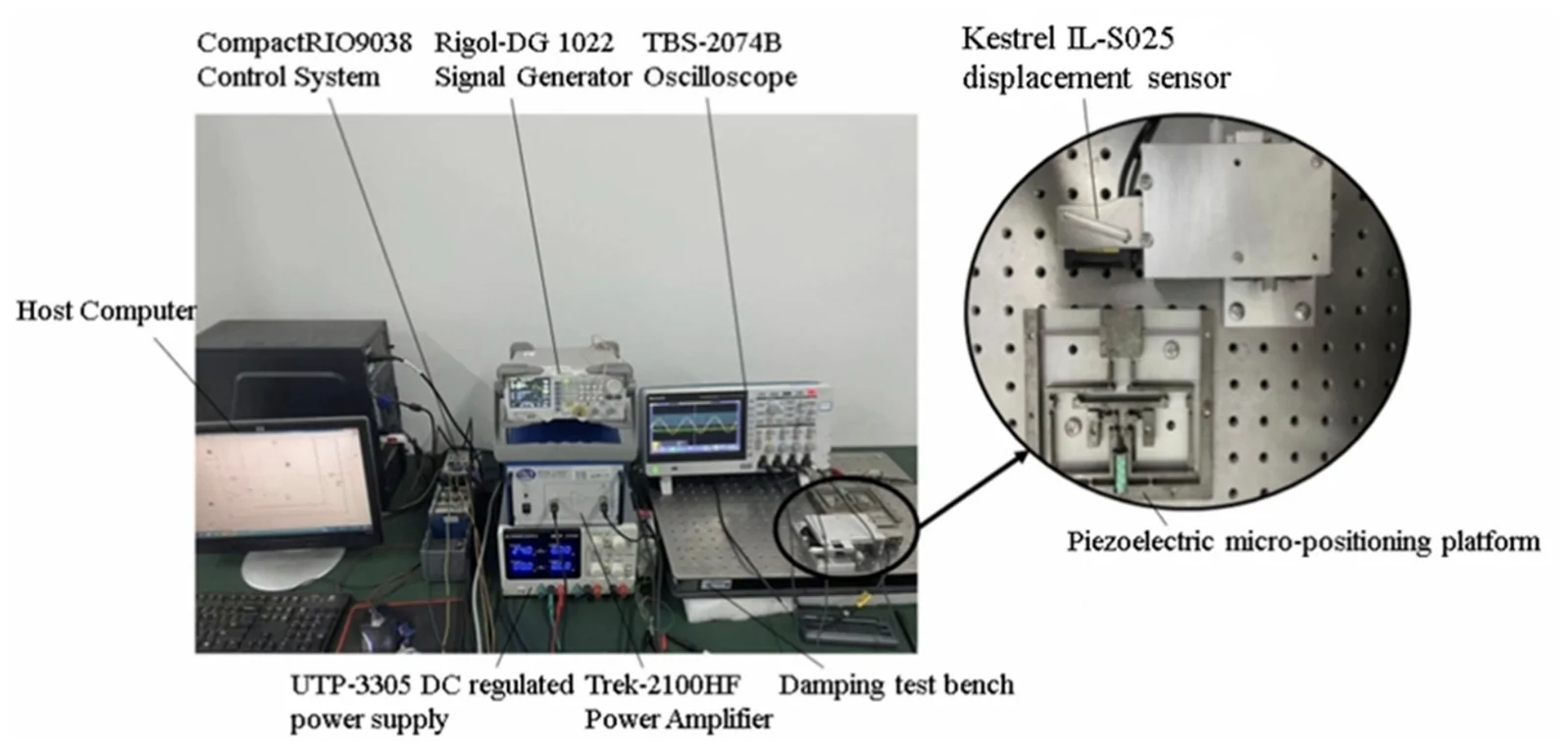
Delay experiment system based on piezoelectric micro-positioning stage.

The hysteresis test bench for the piezoelectric flexure micro-positioning stage consists of actuation, control, sensing and auxiliary instrument modules. The micro-positioning stage with piezoelectric actuator is mounted on a vibration-isolation stage as shown in Figure 23. A power amplifier (Type Trek-2100HF, Trek Inc., Medina, NY, USA), which amplifies control signals output from real-time controller (NI CompactRIO9038, National Instruments, Austin, TX, USA) to drive the piezoelectric actuator. A host computer completes algorithm deployment and data recording. A Keyence IL-S025 laser displacement sensor (Keyence Corporation, Osaka, Japan) provides nanometer-level real-time position feedback for hysteresis characterization and closed-loop control. A Rigol-DG1022 arbitrary waveform generator (Rigol Technologies, Beijing, China) and TBS-2074B digital oscilloscope (Tektronix, Beaverton, OR, USA) are used for excitation signal generation and real-time signal monitoring. A UTP-3305 DC regulated power supply supplies (UNI-T Technologies, Dongguan, Guangdong, China) stable power for all electronic devices.

### 4.2. Error Statistical Evaluation Metrics

To quantitatively evaluate the tracking and hysteresis compensation performance of the proposed adaptive KP control strategy, two statistical error analysis indices, RMSE and MAPE, are adopted to quantify the deviation between measured displacement and model predictive output. The statistical calculation formulas are given as Equation (25):

(25) $$\mathrm{RMSE}=\sqrt{\frac{1}{M}\sum_{k=1}^{M}{\left(y_{k}-{\hat{y}}_{k}\right)}^{2}},\mathrm{MAPE}=\frac{1}{M}\sum_{k=1}^{M}\left|\frac{y_{k}-{\hat{y}}_{k}}{y_{k}}\right|\times 100\%$$

where $y_{k}$ is the real measured displacement at the *k*-th sampling point, ${\hat{y}}_{k}$ represents the predicted output of the adaptive KP control framework proposed, and M denotes the total number of sampling data. RMSE reflects the overall absolute tracking error, while MAPE evaluates the average relative error percentage, which can eliminate the interference caused by different displacement amplitudes.

To further highlight the superiority of the proposed lightweight KP adaptive inverse control, we summarize the tracking accuracy indicators of several representative piezoelectric hysteresis compensation methods from recent published literature, as listed in Table 3. All evaluation metrics adopt the RMSE and MAPE statistical criteria defined in Equation (25) for fair horizontal comparison.

### 4.3. Software Implementation

**Figure 24:**
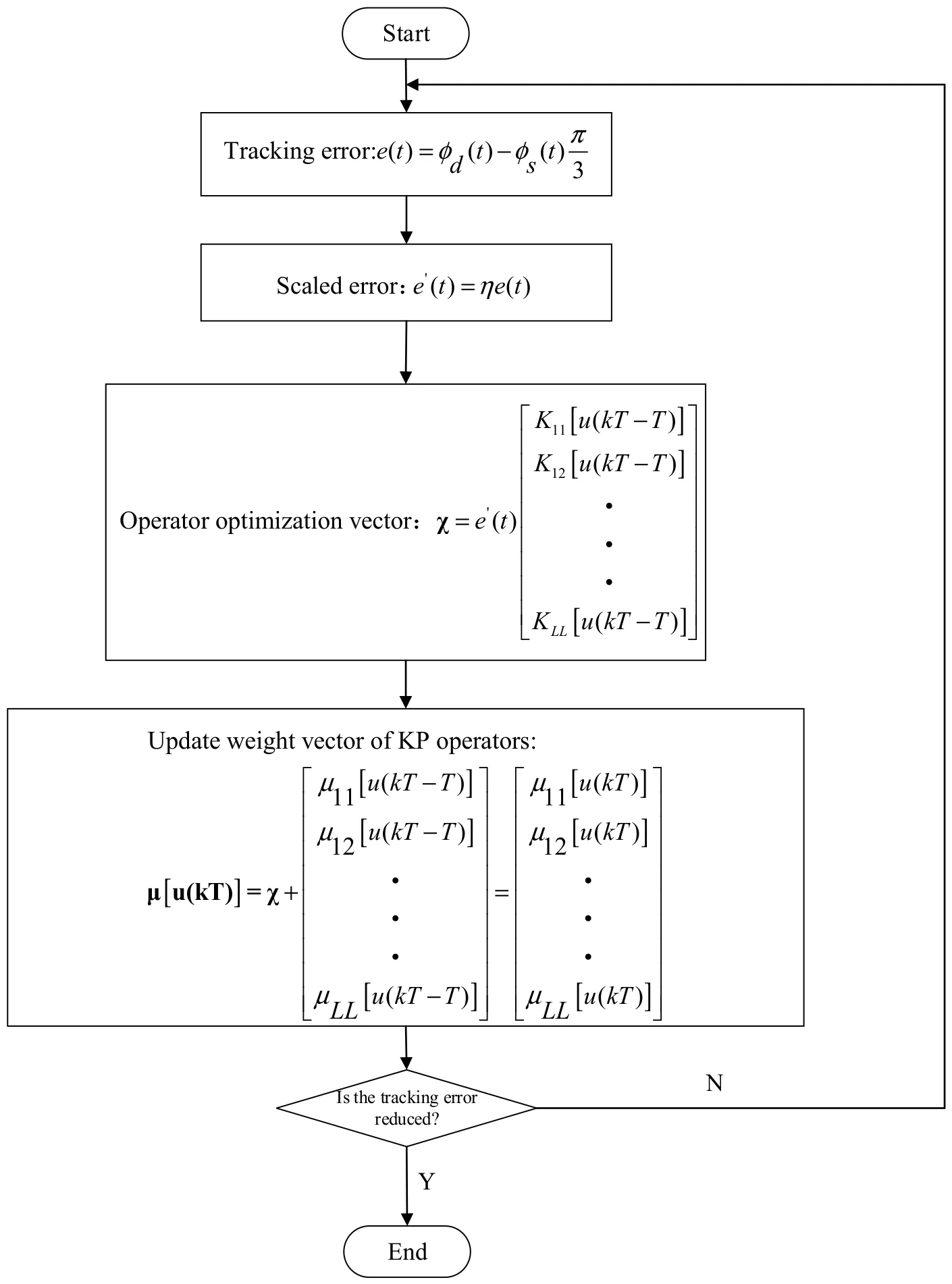
Flowchart for online weight updating of KP operators.

Different from traditional feedback control systems, the adaptive inverse control system combines adaptive inverse control and adaptive feedback control strategies. By online identification of KP model parameters, the inverse model of the KP hysteresis model can be derived. The original KP model is employed as the controller, and the obtained KP inverse model serves as feedforward compensation to realize the inverse hysteresis process. The output voltage calculated by the inverse model is applied to the piezoelectric actuator to generate the forward hysteresis response. The forward and inverse hysteresis effects counteract each other to suppress hysteresis nonlinearity, so that the measured displacement of the piezoelectric actuator gradually converges to the desired displacement. The measured displacement signal is fed back to the control loop, and the learning rate α is set as the feedback gain to construct a closed-loop control system for the KP model. Continuous real-time tuning of model and controller parameters is carried out to achieve satisfactory hysteresis compensation performance. The closed-loop iterative procedure for tracking error calculation and KP operator weight updating is presented in Figure 24. This iterative algorithm is integrated into the adaptive inverse control system to complete online real-time adjustment of KP model weight parameters.

The real-time online weight updating module built into the feedback loop can continuously modify the weight coefficients of each KP operator according to real-time position deviation, which fundamentally avoids the gradual deterioration of compensation accuracy caused by fixed offline model parameters, and the online weight adjustment logic adopted in this paper draws on the adaptive parameter identification framework proposed in Reference [23].

**Figure 25:**
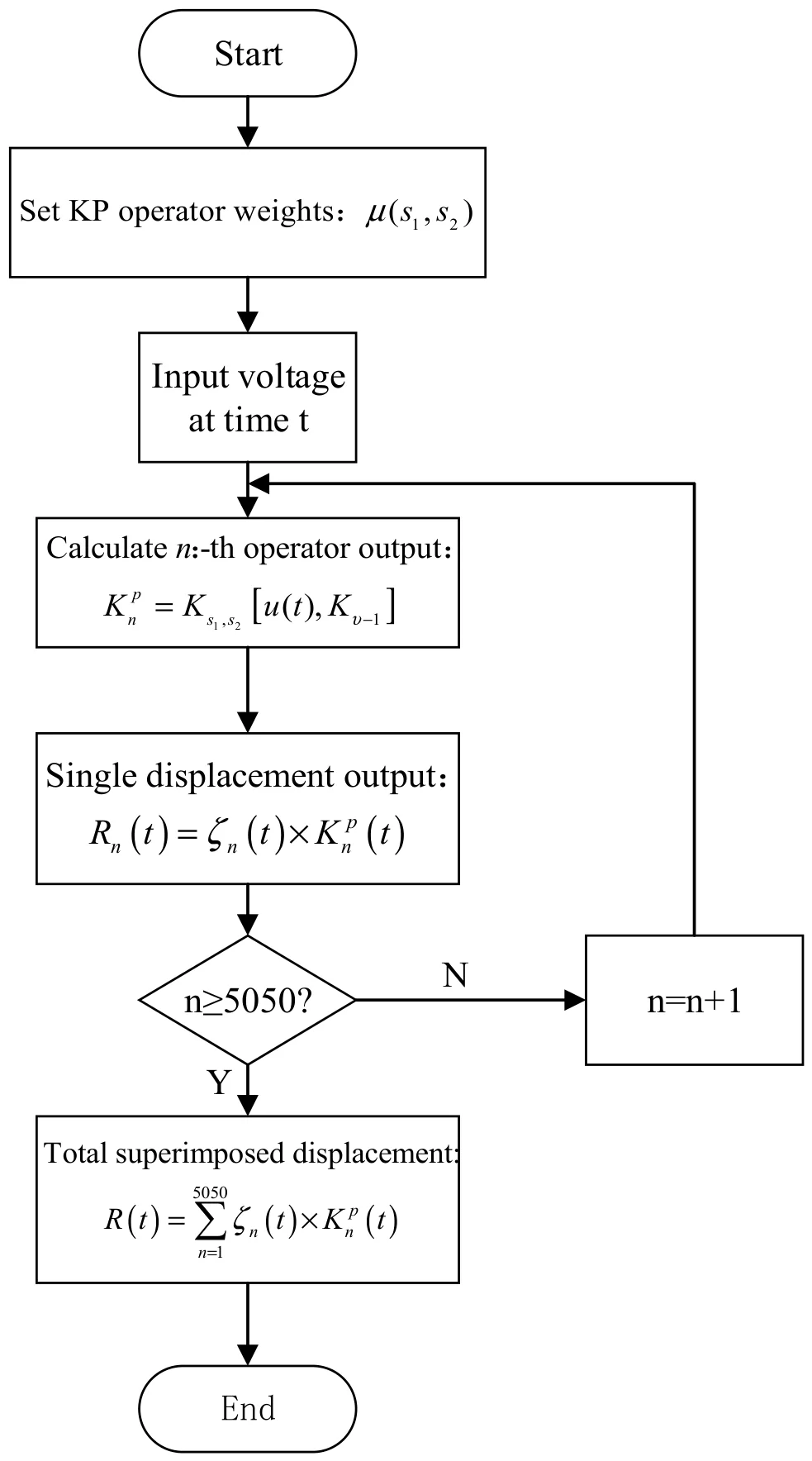
Flowchart for superposition calculation of KP operators.

The KP operator is composed of the slope parameter a, the input of the previous moment of the driving signal, the input of the driving signal, threshold $s_{1}$, $s_{2}$, and the output of the KP operator at the previous moment K(u(t−1)). 5050 KP operators are extracted from the discretized database, with the initial weights set to 1. The output of each operator is calculated, and it is determined whether it meets the required number of operators. If not, the KP operator is incremented, and this process repeats until the required number is reached. If it does meet the requirement, the 5050 KP operators are weighted and superimposed to obtain the output of the KP model. The superimposition program diagram of the KP operator is shown in Figure 25.

### 4.4. Results and Comparative Analysis

**Figure 26:**
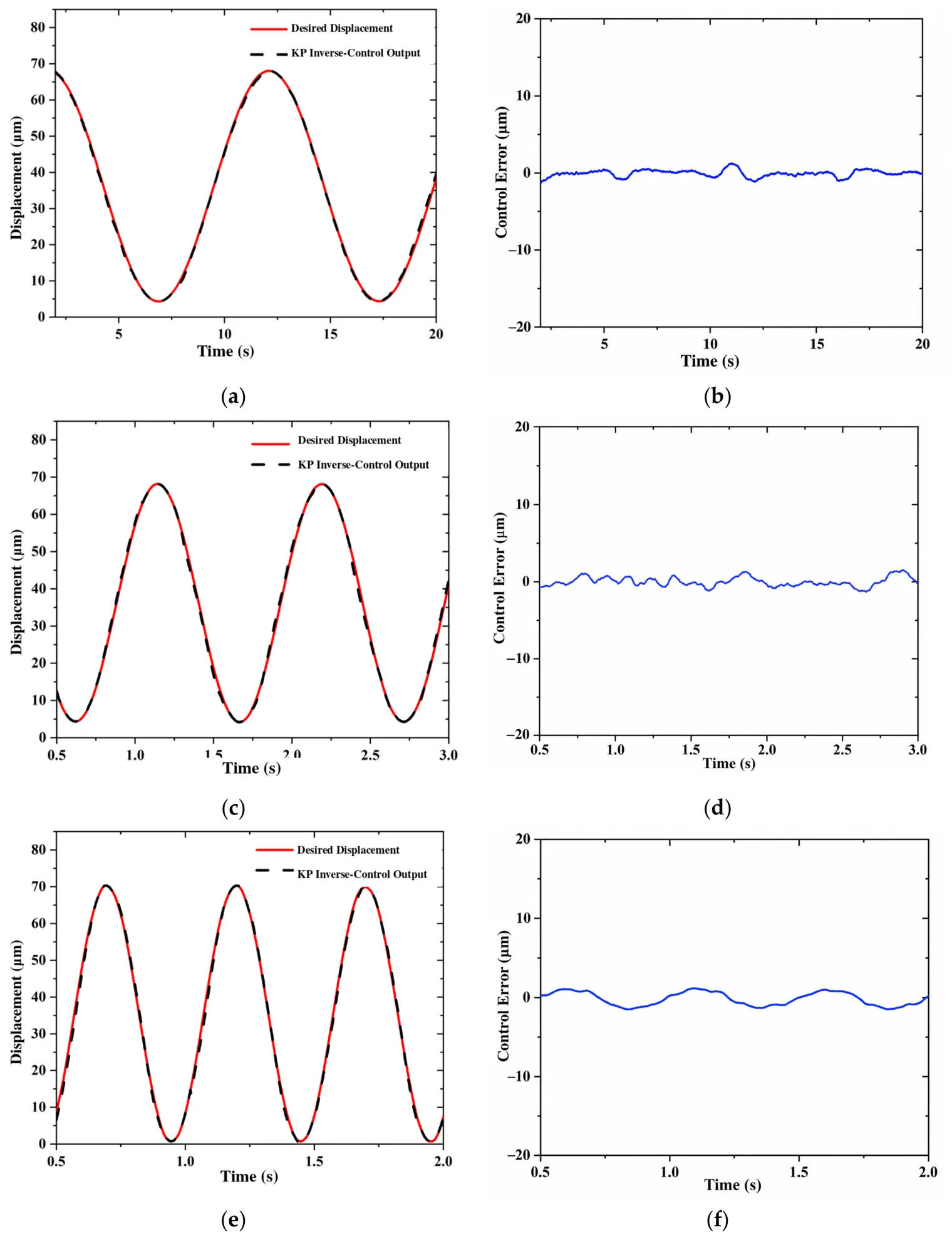
The tracking curve and error curve diagrams of KP adaptive inverse control under sinusoidal drive. (**a**) 0.1 Hz expected displacement and model displacement tracking curve; (**b**) 0.1 Hz error curve; (**c**) 1 Hz expected displacement and model displacement tracking curve; (**d**) 1 Hz error curve; (**e**) 2 Hz expected displacement and model displacement tracking curve; (**f**) 2 Hz error curve.

By observing Figure 26, compared with the model-free control and the KP model control, the KP adaptive inverse control system has a good compensation effect on the lag nonlinearity and significantly reduces the control error. As the driving voltage frequency increases, the control error also increases, but it remains at a relatively low level. From the tracking curve diagram in Figure 26, the tracking performance of the model-free control and the KP model control is poor, and they cannot accurately describe the lag nonlinearity phenomenon of the piezoelectric micro-positioning stage. However, the KP adaptive inverse control can precisely track the displacement output of the piezoelectric micro-positioning stage, and the control error under different voltage frequencies is significantly smaller than that of the model-free control and the KP model control, verifying the effectiveness of the KP adaptive inverse control in the piezoelectric micro-positioning stage. Each group of fixed-frequency sine wave tracking experiments was repeated more than five times under the same test conditions to verify the repeatability of the proposed adaptive controller. A large number of raw data sets were obtained through repeated measurements. Figure 26 only shows the most representative tracking curves and error curves for intuitive comparison.

The above tracking curves qualitatively reflect the superior compensation performance of the proposed scheme against model-free and offline KP control. To further quantitatively highlight the advantages of this method over mainstream rate-dependent KP algorithms, Table 4 lists the statistical RMSE and MAPE of all comparative strategies under 0.1 Hz, 1 Hz and 2 Hz sinusoidal signals. Conventional rate-dependent KP methods introduce extra dynamic correction terms to handle frequency-varying hysteresis, which inevitably raises computational overhead and slows down convergence speed. In contrast, the proposed KP adaptive inverse control only updates operator weights via real-time displacement feedback without complex auxiliary dynamic equations, achieving faster convergence and more stable tracking performance across all test frequencies. As shown in Table 4, the proposed strategy yields the minimum tracking error at every excitation frequency, which clearly demonstrates that the approach overcomes the high computation cost and weak adaptability bottlenecks of existing rate-dependent KP variants.

**Figure 27:**
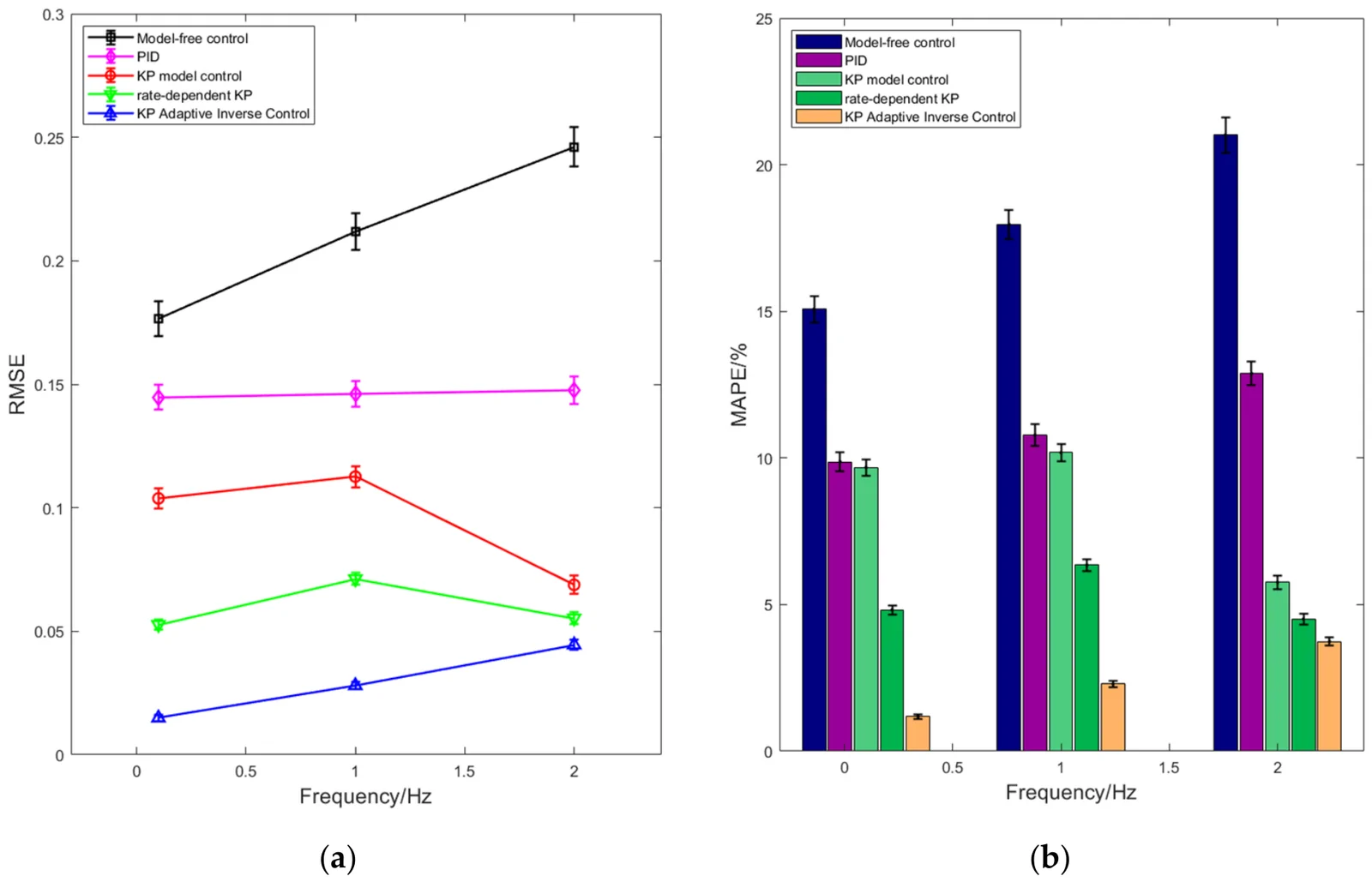
Comparison of tracking error performance of various control algorithms under different excitation frequencies. (**a**) Line chart of root mean square error comparison; (**b**) Average absolute percentage error bar chart.

To more intuitively reflect the hysteresis compensation performance of KP adaptive inverse control, the RMSE line chart and MAPE bar chart of model-free control, KP model control and KP adaptive inverse control are plotted in Figure 27. All RMSE and MAPE data shown in the figure are expressed as mean ± standard deviation from repeated trials, which can effectively verify the repeatability and robustness of each control strategy.

As shown in Figure 27, when the frequency of the sinusoidal excitation increases from 0.1 Hz to 2.0 Hz, the tracking errors of all five control strategies also increase. This is due to the fact that piezoelectric ceramics exhibit stronger lag nonlinear characteristics at higher frequencies. At all tested frequencies, the error magnitudes of the five schemes show a consistent ranking: no-model control > PID > KP model control > rate-dependent KP > KP adaptive inverse control. Among them, the no-model control without lag compensation has the largest RMSE and MAPE values; while PID can only achieve limited error suppression because it cannot model the lag characteristics. The rate-dependent KP model, by capturing the lag effect related to the rate, demonstrates superior compensation accuracy compared to the basic KP model. Among all comparison methods, the proposed KP adaptive inverse control achieves the smallest RMSE and MAPE throughout the frequency range, indicating its excellent lag compensation accuracy and good frequency adaptability. At the same time, its error stripes are the shortest, further verifying the excellent repeatability of this method and its strong robustness to lag disturbances caused by frequency changes.

**Figure 28:**
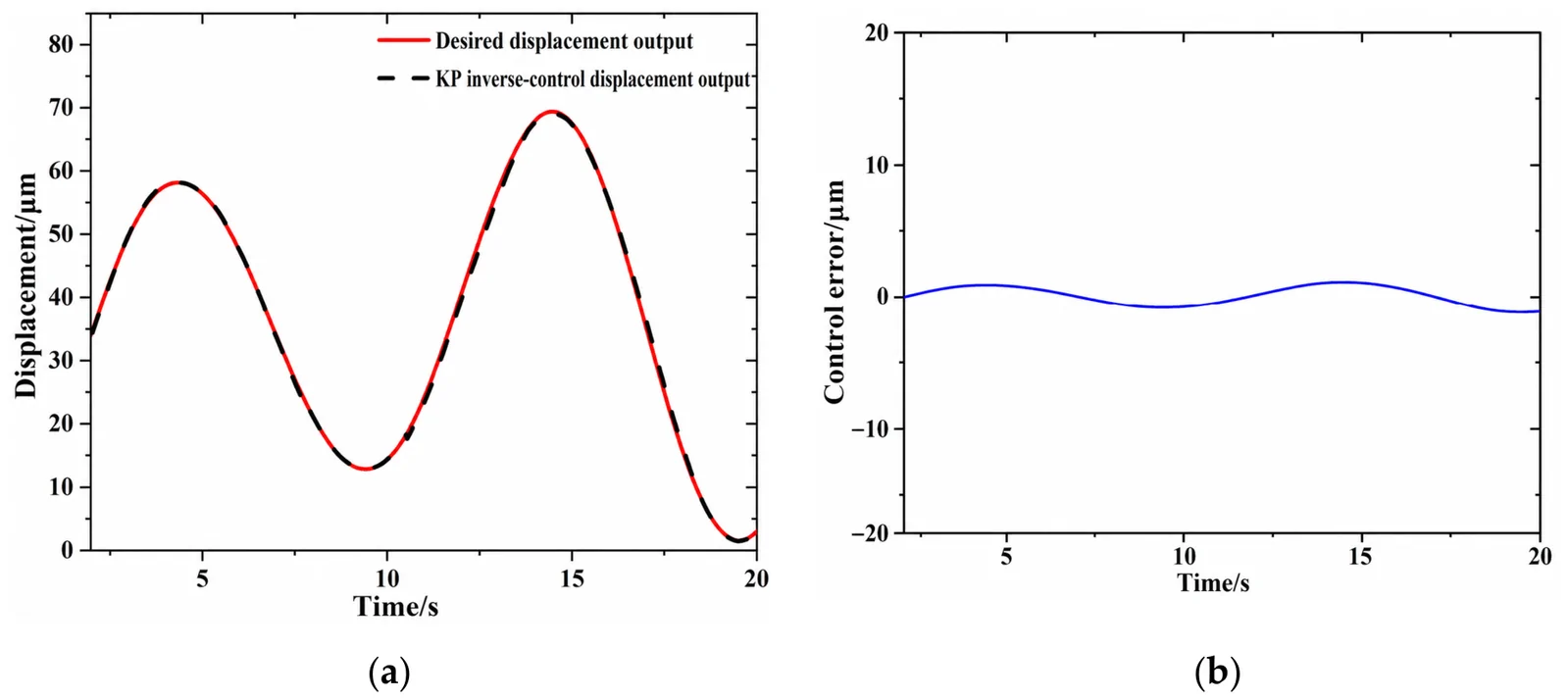

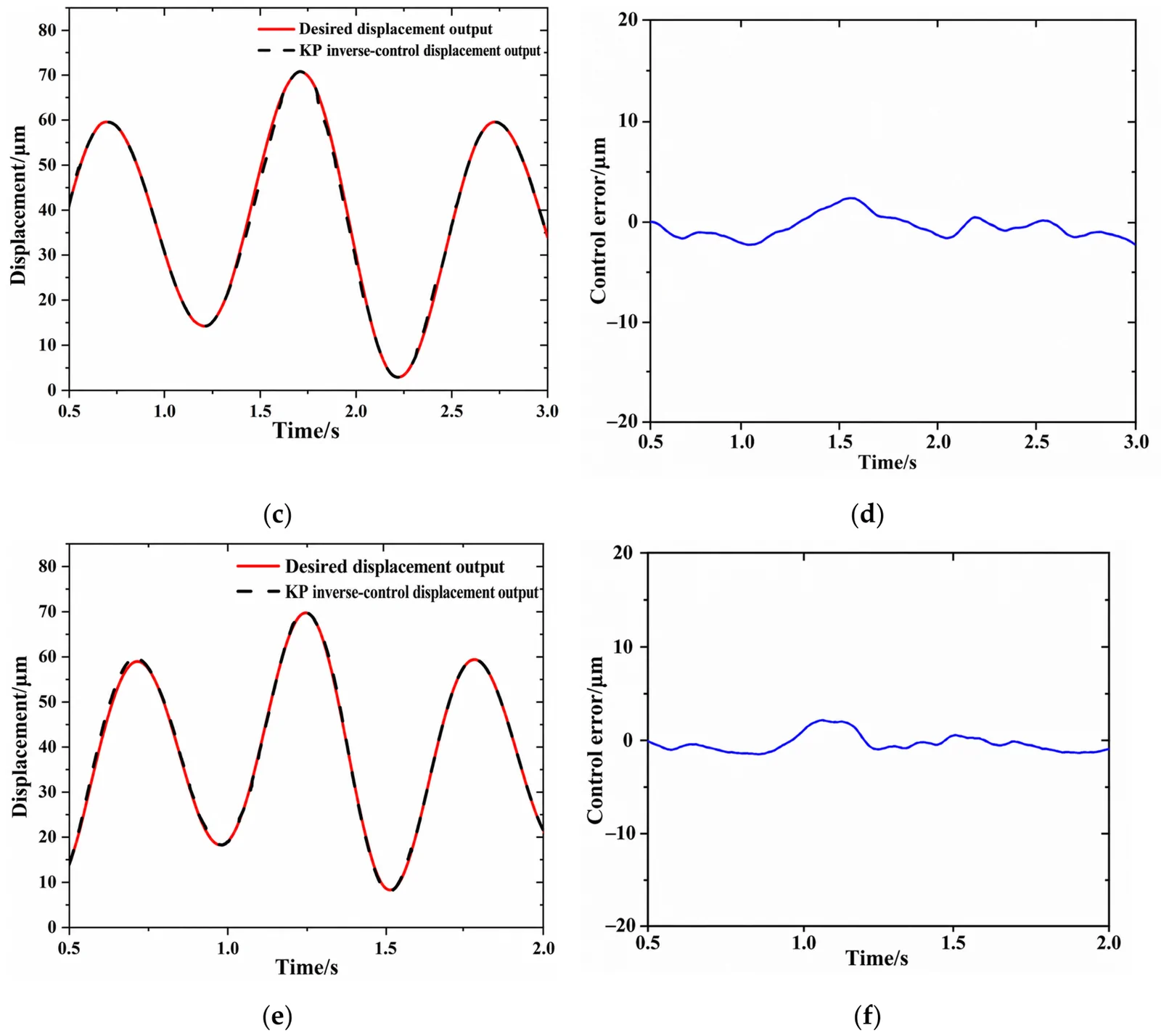
The tracking curve and error curve diagrams of KP adaptive inverse control under ampli-tude-sinusoidal driving. (**a**) 0.1Hz expected displacement and model displacement tracking curve; (**b**) 0.1Hz error curve; (**c**) 1Hz expected displacement and model displacement tracking curve; (**d**) 1Hz error curve; (**e**) 2Hz expected displacement and model displacement tracking curve; (**f**) 2Hz error curve.

From Figure 28, it can be observed that at a frequency of 0.1 Hz, the fitting degree between the displacement output of KP adaptive inverse control and the expected displacement output is the highest. As the driving voltage frequency increases, the fitting degree between the model displacement and the expected displacement gradually decreases. However, compared to the model-free control and the KP model control, KP adaptive inverse control has a good compensation effect for the hysteresis nonlinearity, and the fitting degree remains at a relatively high level. From the tracking curve diagram in Figure 28, it can be concluded that KP adaptive inverse control has excellent tracking performance, accurately fitting the displacement output of the piezoelectric micro-positioning stage, further verifying the universality of KP adaptive inverse control under complex waveform driving. Each group of variable amplitude sine wave tracking experiments was repeated more than five times under the same test conditions to verify the repeatability of the proposed adaptive controller. A large number of raw data sets were obtained through multiple measurements. Figure 28 only shows the most representative and valuable tracking and error curves for intuitive comparison.

The above tracking curves qualitatively reflect the superior compensation performance of the proposed scheme compared to the model-free and offline KP control. To further quantitatively highlight the advantages of this method over the mainstream rate-dependent KP algorithms, Table 5 lists the statistical RMSE and MAPE of all comparison strategies under 0.1 Hz, 1 Hz, and 2 Hz amplitude-sinusoidal signals. The traditional rate-dependent KP methods introduce additional dynamic correction terms to handle the lag effect of frequency changes, which inevitably increases the computational cost and reduces the convergence speed. In contrast, the proposed KP adaptive inverse control method only updates the operator weights through real-time displacement feedback and does not require complex auxiliary dynamic equations, thus achieving faster convergence speed and more stable tracking performance at all test frequencies. As shown in Table 5, the proposed strategy can achieve the smallest tracking error at each excitation frequency, clearly indicating that this method overcomes the high computational cost and insufficient adaptability bottlenecks of the existing rate-dependent KP variants.

**Figure 29:**
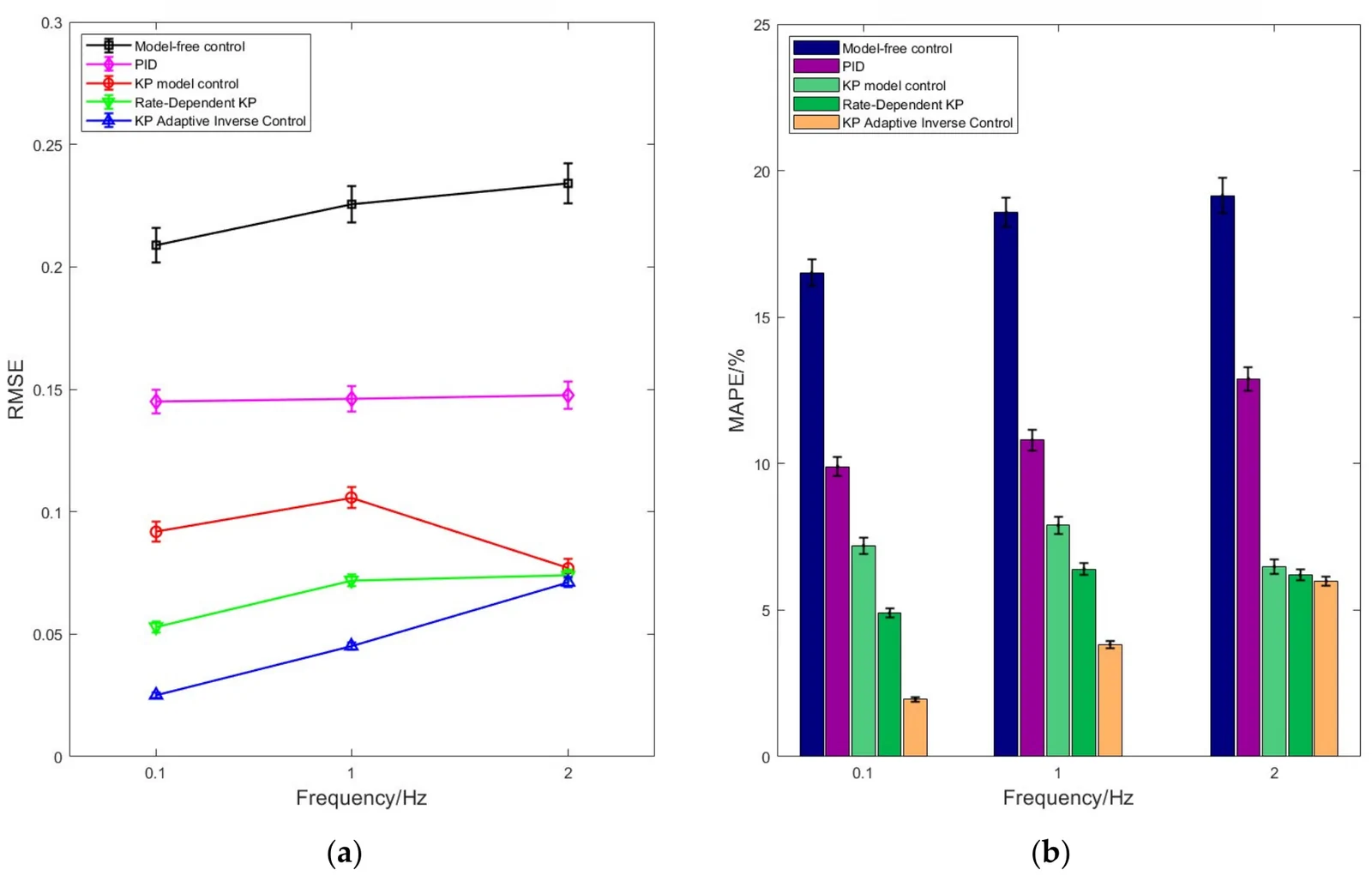
Comparison of tracking error performance of various control algorithms under different excitation frequencies. (**a**) Line chart of root mean square error comparison; (**b**) Average absolute percentage error bar chart.

To more intuitively reflect the hysteresis compensation performance of KP adap-tive inverse control, the RMSE line chart and MAPE bar chart of model-free control, KP model control and KP adaptive inverse control are plotted in Figure 29. All RMSE and MAPE data shown in the figure are expressed as mean ± standard deviation from repeated trials, which can effectively verify the repeatability and robustness of each control strategy.

It can be clearly seen from Figure 29 that the tracking errors of all five control schemes generally increase as the sinusoidal excitation frequency rises from 0.1 Hz to 2 Hz due to the aggravated rate-dependent hysteresis nonlinearity of piezoelectric ceramics. At all tested frequencies, the error magnitude always follows the consistent order: Model-free control > PID > KP model control > Rate-Dependent KP > KP Adaptive Inverse Control. Model-free control without hysteresis compensation yields the largest normalized RMSE and MAPE values, while PID only achieves limited error reduction without hysteresis modeling. The Rate-Dependent KP model obtains better compensation accuracy than the basic KP model by capturing frequency-related hysteresis characteristics. Among all comparative methods, the proposed KP Adaptive Inverse Control achieves the minimum RMSE and MAPE across the full frequency range, which demonstrates its superior hysteresis compensation precision and strong adaptability to varying driving frequencies. Furthermore, its shortest error bars among all control strategies further verify its excellent test repeatability and robustness against frequency-varying hysteresis disturbances.

## 5. Conclusions

This study proposes a hysteresis compensation approach combining the Krasnosel’skii-Pokrovskii (KP) hysteresis model and adaptive inverse control for piezoelectric flexure micro-positioning stages. The main contributions are summarized as follows:

(1) Structural design and mechanical validation of a symmetric two-stage flexure-amplified piezoelectric flexure micro-positioning stage. A single-degree-of-freedom stage with symmetric two-stage lever amplification and straight circular flexure hinges is developed. ANSYS finite element simulations are conducted to verify structural rationality, mechanical strength, and displacement amplification performance, confirming its suitability for precision positioning applications.
(2) High-precision hysteresis modeling using the KP operator-based framework. A Krasnosel’skii-Pokrovskii hysteresis model is established to accurately characterize the rate-dependent and asymmetric hysteresis behavior of piezoelectric actuators. Compared with conventional phenomenological models, the proposed KP model achieves a favorable balance between modeling accuracy and computational efficiency.
(3) Development of an adaptive inverse control strategy for real-time hysteresis suppression. By integrating KP model-based feedback-driven online parameter updating, the proposed control scheme overcomes the limitations of offline-identified hysteresis models and effectively mitigates the influence of hysteresis nonlinearity, external disturbances, and parameter variations.
(4) Experimental validation and performance enhancement. Comparative experiments under sinusoidal and variable-amplitude sinusoidal excitation signals demonstrate that the proposed method reduces the root-mean-square error (RMSE) by 7.41–85.65% and the mean absolute percentage error (MAPE) by 7.56–87.81%, significantly improving positioning accuracy and system robustness.

In future work, we will replace the current low-computing NI CompactRIO-9038 controller with high-performance real-time hardware to carry out wide-frequency-band tracking experiments above 2 Hz. Systematic performance tests including anti-disturbance robustness, positioning repeatability and nanometer resolution will be completed. Meanwhile, intelligent optimization algorithms will be integrated to improve the adaptability of the control scheme under variable frequencies, amplitudes and external loads, and the cross-coupling error and long-term performance degradation of multi-degree-of-freedom stages will be further studied to expand the engineering application range of the proposed hysteresis compensation method.

## Figures and Tables

**Table 1:** Micro-positioning stage input and output displacement comparison diagram.

Input Input displacement/μm	1	5	10	15	20	25	30
Output Output displacement/μm	3.20	15.99	31.98	47.98	63.97	79.96	95.95

**Table 2:** Inherent frequency of micro-positioning stage.

Degree	1	2	3	4	5	6
Natural frequency/Hz	268.24	374.26	684.17	730.36	914.19	932.26

**Table 3:** Performance comparison of state-of-the-art hysteresis-compensation schemes for piezoelectric micro-positioning stage.

Reference	Control Method	Hysteresis Model	Range (μm)	Maximum Error (μm)	Maximum Relative Error (%)
[22]	Feedforward compensation based on dynamic Prandtl–Ishlinskii model + PID feedback hybrid control	Dynamic Prandtl–Ishlinskii model	100	2.13	2.13
[20]	Feedforward compensation based on inverse Duhem operator + adaptive sliding mode hybrid control	Inverse Duhem operator + Generalized Regression Neural Network	17,400	49.4	0.28
[23]	Adaptive tracking control based on Krasnosel’skii-Pokrovskii operator	Krasnosel’skii-Pokrovskii operator model	55	0.53	0.96
[24]	Four-layer modified neural network with adaptive learning	Modified Prandtl–Ishlinskii model with dead-zone operator + neural network	100	12.61	12.61

**Table 4:** Comparison of control errors under sinusoidal drive.

Frequency/Hz	Model	RMSE	MAPE/%
0.1	without Model	0.1766	15.08
	PID	0.1448	9.87
	KP model	0.1038	9.68
	Rate-Dependent KP	0.0527	4.82
	KP Adaptive inverse model	0.0149	1.18
1	without Model	0.2119	17.97
	PID	0.1461	10.78
	KP model	0.1126	10.18
	rate-dependent KP	0.0712	6.35
	KP Adaptive inverse model	0.0280	2.29
2	without Model	0.2460	21.02
	PID	0.1476	12.88
	KP model	0.0688	5.76
	rate-dependent KP	0.0553	4.51
	KP Adaptive inverse model	0.0445	3.74

**Table 5:** Comparison of control errors under sinusoidal drive of amplitude variation.

Frequency/Hz	Model	RMSE	MAPE/%
0.1	Model-free	0.2089	16.51
	PID	0.1450	9.90
	KP model	0.0918	7.20
	Rate-Dependent KP	0.0530	4.90
	KP Adaptive inverse model	0.0251	1.96
1	Model-free	0.2255	18.58
	PID	0.1460	10.80
	KP model	0.1058	7.89
	Rate-Dependent KP	0.0720	6.40
	KP Adaptive inverse model	0.0452	3.83
2	Model-free	0.2341	19.15
	PID	0.1475	12.90
	KP model	0.0769	6.48
	Rate-Dependent KP	0.0740	6.20
	KP Adaptive inverse model	0.0712	5.99

## Data Availability

The data that support the findings of this study are available from the corresponding author upon reasonable request.
